# Diel Variation of Biogenic Volatile Organic Compound Emissions- A field Study in the Sub, Low and High Arctic on the Effect of Temperature and Light

**DOI:** 10.1371/journal.pone.0123610

**Published:** 2015-04-21

**Authors:** Frida Lindwall, Patrick Faubert, Riikka Rinnan

**Affiliations:** 1 Terrestrial Ecology section, Department of Biology, University of Copenhagen, Copenhagen, Denmark; 2 Center for permafrost, Department of Geoscience and Natural resource Management, University of Copenhagen, Copenhagen, Denmark; 3 Chaire en éco-conseil, Département des sciences fondamentales, Université du Québec à Chicoutimi, Chicoutimi, Canada; University of Tennessee, UNITED STATES

## Abstract

Many hours of sunlight in the midnight sun period suggest that significant amounts of biogenic volatile organic compounds (BVOCs) may be released from arctic ecosystems during night-time. However, the emissions from these ecosystems are rarely studied and limited to point measurements during daytime. We measured BVOC emissions during 24-hour periods in the field using a push-pull chamber technique and collection of volatiles in adsorbent cartridges followed by analysis with gas chromatography- mass spectrometry. Five different arctic vegetation communities were examined: high arctic heaths dominated by *Salix arctica* and *Cassiope tetragona*, low arctic heaths dominated by *Salix glauca* and *Betula nana* and a subarctic peatland dominated by the moss *Warnstorfia exannulata* and the sedge *Eriophorum russeolum*. We also addressed how climate warming affects the 24-hour emission and how the daytime emissions respond to sudden darkness. The emissions from the high arctic sites were lowest and had a strong diel variation with almost no emissions during night-time. The low arctic sites as well as the subarctic site had a more stable release of BVOCs during the 24-hour period with night-time emissions in the same range as those during the day. These results warn against overlooking the night period when considering arctic emissions. During the day, the quantity of BVOCs and the number of different compounds emitted was higher under ambient light than in darkness. The monoterpenes α-fenchene, α -phellandrene, 3-carene and α-terpinene as well as isoprene were absent in dark measurements during the day. Warming by open top chambers increased the emission rates both in the high and low arctic sites, forewarning higher emissions in a future warmer climate in the Arctic.

## Introduction

Biogenic volatile organic compounds (BVOCs) have been frequently studied in temperate and boreal ecosystems (e.g. [1–5]). Arctic environments, however, are scarcely studied and have been limited to point measurements during daytime [6].

The global emission of BVOCs from terrestrial ecosystems, estimated to be 700–1000 Tg C yr^-1^ [7], contributes to physical and chemical properties in the atmosphere. BVOCs may prolong the lifetime of methane in the atmosphere, affect the concentration of near ground ozone and play a role in cloud formation processes [8]. Thus, BVOCs are important due to their reactivity and contribution to atmospheric chemistry and their unknown feedbacks to climate change [9,10].

Terpenoids, a large group of BVOCs, include hemiterpenes (e.g. isoprene), monoterpenes (MTs) and sesquiterpenes (SQTs). Isoprene, globally the most abundant individual BVOC [7], is not stored in plants, but is emitted directly from *de novo* synthesis, which is dependent on products from photosynthesis [11]. In contrast, emissions of MTs and SQTs can derive from both *de novo* synthesis and storage pools, such as glandular trichomes and resin ducts [11,12]. The emission of *de novo* synthesized compounds is light and temperature dependent while that of compounds released from storage pools is mainly dependent on leaf temperature [7]. Thus, emission of BVOCs often peaks during midday and decreases with decreasing light and temperature (see e.g. McKinney et al. [13]). The emission from arctic ecosystems, with low temperature, low foliar density and low solar angle, has been previously estimated to be minimal [14]. However, Schollert et al. [6] reported significant BVOC emission rates for high arctic vegetation (20–60 μg m^-2^ h^-1^), up to the same order of magnitude as the emissions from subarctic vegetation (45 μg m^-2^ h^-1^) [15].

Arctic plants are well adapted to the short growing season, and photosynthetic activity in arctic evergreen species occurs even under the snow cover in spring time [16]. The capacity to produce isoprene develops fast in these systems; the onset of isoprene emission has been shown to occur already at 100°D (degree days; number of temperature degrees above a threshold accumulated for each day) in a subarctic wetland [17] compared to boreal woody ecosystems where the onset occurs at 200–600°D [18,19]. It is evident that plants in arctic biomes are active and produce and emit BVOCs even though the mean air temperature is rather low. However, there is great uncertainty about the amount of BVOCs released from arctic ecosystems and the physiological and environmental parameters affecting the emission rate. The amount and composition of BVOCs released from the Arctic, where climate change is more rapid than in any other biomes in the world [9], needs greater attention as it is likely to be of increasing importance to the global BVOC budget.

A diel variation in BVOC emissions has been reported for several different environments and plant species, for example for a Mediterranean mixed oak forest [20], for Eucalyptus trees in a greenhouse [21] and in a laboratory experiment [22] and for Scots pine in a boreal conifer forest [23]. To date, no study has reported on the diel variation of BVOC emissions from ecosystems in the Arctic, where there is solar radiation during night-time in the midnight sun period, the time of the year during which the sun never sets below the horizon. Rinne et al. [24] have reported that sub-canopy isoprene concentrations in a boreal forest peak just before midnight, and suggested that the late peak is due to the more sunlight hours in the high compared to mid-latitudes, where isoprene peaks at mid-day [25]. An unusual high particle growth rate during the night above a subarctic wetland [26] may indicate that there are significant BVOC emissions during night in this area, knowing that BVOCs contribute to particle formation [7]. It is likely that the unique light conditions in the Arctic have an effect on the diel variation of BVOC emissions and the potential night-time emissions cannot be ignored in emission budgets and models. There is an urgent need for researchers to obtain reliable emission estimates and to improve the models used today.

No studies have reported on the effect of warming on the diel variation of BVOC emissions in the arctic ecosystems. It has been estimated that climate warming during the last 30 years has increased global BVOC emissions by approximately 10% and a further temperature increase of 2–3°C could increase emissions by an additional 30–45% [27]. Tiiva et al. [28] have reported an increase in isoprene emission by as much as 83% due to experimental warming and Faubert et al. [29] have shown that climate warming may have more impact on the daytime BVOC emissions from subarctic dry heath than what has been suggested by commonly used temperature dependency models [14,30].

Our aim was to elucidate the diel variations of BVOC emissions using field measurements in five vegetation communities from the Subarctic, Low Arctic and High Arctic. We expected significant BVOC emissions during the day but also during the night-time due to the midnight sun. We also expected some nigh-time emissions of lipophilic BVOCs, such as non-oxygenated MTs, from storage pools, because they can diffuse through the cuticle even if stomata close. We also aimed to assess the importance of climate warming on the rates and diel variation of BVOC emissions. The effect of warming by open top chambers (OTCs) during the snow free period correlates with the amount of photosynthetic active radiation (PAR) and has been reported to be larger during the daytime than night-time [31]. Thus, it was expected that the effect of OTC warming on the BVOC emissions would be more pronounced during the day when the solar angle is higher than during the night. Finally, we aimed to address the immediate effect of light by comparing emissions sampled under ambient light and in complete darkness. We hypothesized that darkness would decrease the emission rate of compounds that derive directly from *de novo* synthesis due to a halt in production, as well as hydrophilic compounds deriving from both *de novo* synthesis and storage pools, for example oxygenated MTs which cannot diffuse through the cell membranes, due to stomatal closure. However, we did not expect that the light manipulation would have an effect on emission from pools in specialized storage structures.

## Materials and Methods

### Site description and experimental design

**Fig 1:**
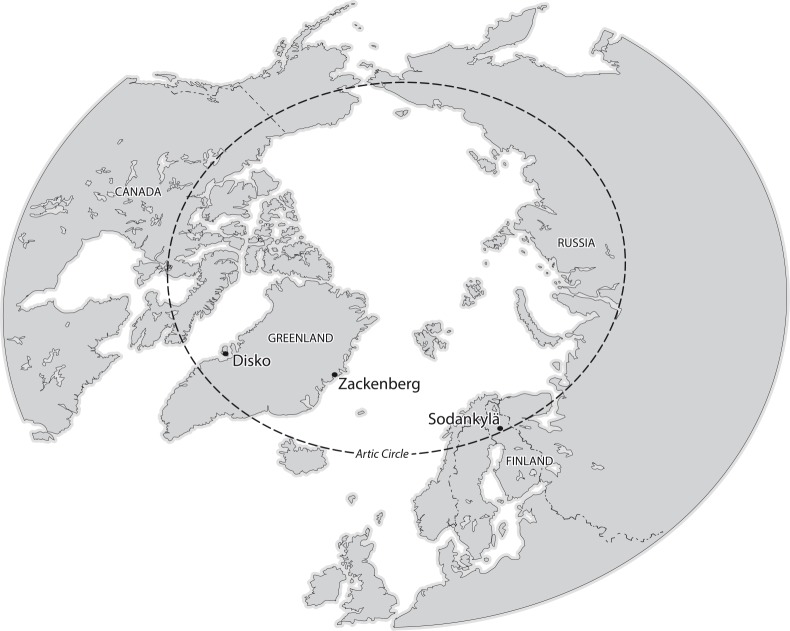
Map showing the three study sites in the Arctic area: the high arctic Zackenberg valley (NE Greenland; 74°30’N / 21°00’W), the low arctic Disko Island (W Greenland; 69°14’N / 53°32’W) and the subarctic Sodankylä (N Finland 67°22’N / 26°38’E).

The 24-hour measurements were performed in high arctic (Zackenberg valley, NE Greenland; 74°30’N / 21°00’W), low arctic (Disko Island, West Greenland; 69°14’N / 53°32’W) and subarctic (Sodankylä, Northern Finland; 67°22’N / 26°38’E) study sites (Fig 1). Here the High Arctic is defined as the northernmost part of the arctic area with open very low-statured vegetation and a mean temperature for the warmest month of ~6°C [6,32]. The Low Arctic has more lush vegetation and a mean temperature for the warmest month of ~8–9°C [32]. The Subarctic is an area immediately south from the true Arctic with monthly mean temperature of more than 10°C for at least one and maximum three months [33].

The high arctic sampling sites were situated in a valley where the mean annual precipitation was 261 mm (84% as snowfall) and the mean annual temperature was -7.8°C (1996–2005 period; [34]). This area experiences midnight sun during a 96-day period between 4 May and 8 August [35]. The sampling was performed in two different heath vegetation communities, one dominated by *Cassiope tetragona* (L) D. Don and another dominated by *Salix arctica* L. For detailed description of the vegetation see S1 Table. In each vegetation community, two controls (C) and two plots warmed (W) by hexagon-shaped open top chambers (OTCs; diameter 120 cm, the International Tundra Experiment, ITEX) acting as open greenhouses, installed in 2007 in a randomized block design, were sampled simultaneously. Another two plots from each treatment were sampled 45 min after the first set. This experimental design with four replicates allowed us to compare the diel BVOC emission patterns between the two vegetation communities and the treatments.

The low arctic site was a heath located close to the ocean with an average annual temperature of -2.9°C and precipitation of 273 mm (1992–2013 period). Midnight sun occurs for a period of 52 days between 26 May and 17 July [35]. The vegetation is overall low statured and dominated by *Salix glauca* L., *Betula nana* L., *Vaccinium uliginosum* L. and *Carex* spp. (S2 Table).

The subarctic site, located in Sodankylä, Northern Finland, was a mesotrophic flark fen on the larger peatland complex Halssiaapa (67°22’N, 26°38’E, 179 m a.s.l.; [36]). The mean annual temperature was -0.4°C between 1981 and 2010, and the mean annual precipitation for the same period was 527 mm [37]. This site has midnight sun for a period of 20 days between 11 June and 1 July [35].The dominant plant species were the moss *Warnstorfia exannulata* (Schimo.) Loeske and the sedge *Eriophorum russeolum* Fr. Ex Hartm. (S3 Table). The measurements took place in the control plots of an experiment assessing long-term effects of enhanced UV-B radiation [36].

One measurement campaign, performed to study the effect of light vs. darkness, took place on a low arctic heath a few kilometers in a westerly direction from the low arctic site described above. The dominant plant species were *V*. *uliginosum*, *Empetrum nigrum* ssp. *hermaphroditum* L. and *C*. *tetragona* (S4 Table). Measurements took place in a one-year-old climate experiment where samples were taken from controls (C) and plots warmed by OTCs (W) similar to the high arctic site.

### Ethics Statement

No endangered or protected species were sampled in this study. The Ecosystem Monitoring Coordination Group at the National Environmental Research Institute, Aarhus University approved access and research activities in the Zackenberg national park, and the board of the Arctic Station those in the protected area on Disko Island. No permits were needed for the subarctic site as the area is not privately-owned or protected.

### BVOC sampling and analysis

The sampling took place every three hours in high- and low arctic sites and every two hours in the subarctic site giving a total of 8 and 12 sampling points during a 24-hour period, respectively. In the High Arctic the *Cassiope*-heath was measured on 24–25 July and the *Salix*-heath on 25–26 July 2013. In the Low Arctic, measurements were conducted on 18–19 June 2013 in two different communities dominated by *S*. *glauca* or *B*. *nana*. The subarctic site was measured on 23–24 July and 5–6 August 2008. Four replicate plots were measured each time for each vegetation community. To study the effect of light vs. darkness during the day BVOC emissions were sampled from four control and four warming plots first under ambient light and then while darkening the measurement chamber by a black cloth. The chamber was darkened for 10 min prior to the start of sampling. The sampling under light and dark conditions was separated by one hour to minimize the stress effect on the plants but to maintain environmental conditions as similar as possible. It took place between 09:00 and 16:00 on 15 June 2013.

An enclosure technique was used to sample BVOCs emitted from the whole ecosystem plot including both vegetation and the underlying soil (see Faubert et al. [36] for the method used in the subarctic site and below for the method used in the high- and low arctic sites). Transparent polycarbonate chambers (thickness 1.5 mm; size for the high and low arctic sites: 22 × 22 cm, height 20 cm; size for the subarctic site: 60 × 60 cm, height 25 cm; Vink Finland, Kerava, Finland) were placed on aluminum chamber bases that were permanently installed in the ground. The chamber base had a groove that was filled with water to seal and ensure that the chamber headspace was airtight. Inside the chambers a fan mixed the air. Incoming air, which was purified by a charcoal filter to remove particles and BVOCs and by a manganese dioxide scrubber to remove ozone, was first directed into the chamber at 1000 ml min^-1^ for 10 min using battery operating pumps, to exchange the ambient air therein. Thereafter the inflow was set to 200 ml min^-1^. This procedure implies that in the beginning of the sampling period, the low BVOC concentration in the chamber may have induced release of BVOCs from storage pools and soil due to the large concentration gradient. However, during the 30 min sampling period, the concentration gradient is likely to diminish and possibly even reverse, suggesting few net effects on release of BVOCs. A stainless steel adsorbent cartridge filled with 150 mg Tenax TA and 200 mg Carbograph 1TD (Markes International Limited, Llantrisant, UK) was attached to the outflow-connection and air was drawn out from the chamber through the cartridge for 30 min at 200 ml min^-1^. The temperature and relative humidity inside the chambers were recorded with a shaded iButton (i-Wire Hygrochron, Maxim Intergrated, San Jose, USA) and the PAR was recorded every minute during the sampling using a S-LIA-M003 PAR sensor connected to a HOBO micro station data logger (H21-002 Onset computers corporation, Boston, USA). After sampling, the cartridges were sealed with Teflon-coated brass caps and stored at 4°C until transport and analysis in Copenhagen, Denmark. The measurement chambers were also removed and cleaned, using paper towels, to eliminate water and possible memory effects from sticky compounds that might have attached on chamber surface. Blank measurements to detect compounds originating from the material used during the measurement or analysis system were performed by covering the chamber base with a pre-cleaned polyethylene terephthalate (PET) film to exclude the soil and vegetation. These measurements were performed *in situ* in ambient conditions.

The BVOCs were analyzed by thermal desorption, at 250°C and cryofocusing at -10°C (UNITY2, Markes, Llantrisant, UK), coupled with an ULTRA autosampler and gas chromatograph-mass spectromer (7890A Series GC coupled with a 5975C inert MSD/DS Performance Turbo EI System, Agilent, Santa Clara, CA, USA), with a HP-5 capillary column (50 m × 0.2 mm, film thickness 0.33 μm). Oven temperature was 40°C for 1 min, then increased by 5°C min^-1^ until 210°C, and finally increased at a rate of 20°C min^-1^ up to 250°C. Helium was the carrier gas and the run time was 45 minutes. BVOCs were grouped into isoprene, MTs, SQTs, other reactive volatile organic compounds (ORVOCs; compounds having a lifetime in the atmosphere less than 24 hours) and other volatile organic compounds (other VOCs; compounds having a lifetime in the atmosphere longer than 24 hours) [14]. The BVOCs were identified using pure liquid standards and according to the mass spectra in NIST library and quantified using pure standards for tricyclene, 2-methylfuran, toluene, nonanal, 2-hexenal, 1-octen-3-ol, bornylacetate, α-pinene, camphene, sabinene, β-myrcene, β-pinene, α-phellandrene, 3-carene, d-limonene, 1,8-cineole, α-terpinene, terpinolene, linalool, camphor, borneol, α-copaene, longifolene, β-caryophyllene, α-humulene, *cis*-3-hexenyl acetate, methyl salicylate and isoprene. Standards in methanol solution were injected in adsorbent cartridges in helium stream. The compound α-pinene was used for quantifying MTs which did not have a pure standard available, 1,8-cineole for oxygenated MTs, α-humulene for SQTs, and toluene for ORVOCs and other VOCs (in the subarctic data, *cis*-3-hexenyl acetate was used for ORVOCs and methyl salicylate for other VOCs). Compounds that had an identification quality in the NIST library of above 85% were included in the dataset. Compounds which were found to originate from the system, i.e. found in blanks, were subtracted from samples. The BVOC emission rates (μg BVOC m^-2^ ground area h^-1^) were calculated as in Faubert et al. [15]. To calculate a daily sum, the hourly emission rates were first multiplied by three in the High and Low Arctic and two in the Subarctic, to represent the emission rates of the three or two-hour periods, respectively. Then, these products were summed to obtain an estimate for an emission rate for a 24-hour period.

### Statistical analyses

For each BVOC group, differences in emissions between time points during the 24-hour period were tested using a one-way ANOVA mixed model computed in the R software (3.0.1, R core team, 2013, package: lme4 version 1.0–5), with time as a fixed factor and plot as a random factor. When an additional factor was introduced, such as the warming treatment in the high arctic data, it was added in the model as a fixed factor, and a two-way ANOVA mixed model was used. If there was a significant difference between time points, a Dunnett’s test was used to compare the different time points against the midday measurement, 12:00 for the high- and low arctic data and 13:00 for the subarctic data. Differences between treatments were also tested separately for all time points and Bonferroni adjusted P-values were used to correct for the multiple comparisons. Student´s t-test was used for testing difference in emission rates between vegetation communities. The effect of warming, by OTCs, on chamber and soil temperature, soil moisture, and differences in daily sum between vegetation types were tested using one-way ANOVA.

The light/dark data were tested using a two-way ANCOVA mixed model, with chamber temperature as a covariate (to account for the unwanted effect of the light treatment on temperature), the light/dark factor and the field treatment (C, W) as fixed factors and plot as a random factor. A Pearson’s correlation test was used for testing correlations between emission rates and environmental parameters (PAR and temperature inside measurement chambers). In general, an α = 0.05 was used, but to reduce the risk of Type II errors, which is rather high due to the limited amount of replicates, we also discuss trends at a 0.05<α<0.1. The data were log-transformed to obtain normal distribution and equal variances.

## Results

### Variation of emissions over 24 hours

#### High Arctic

**Fig 2:**
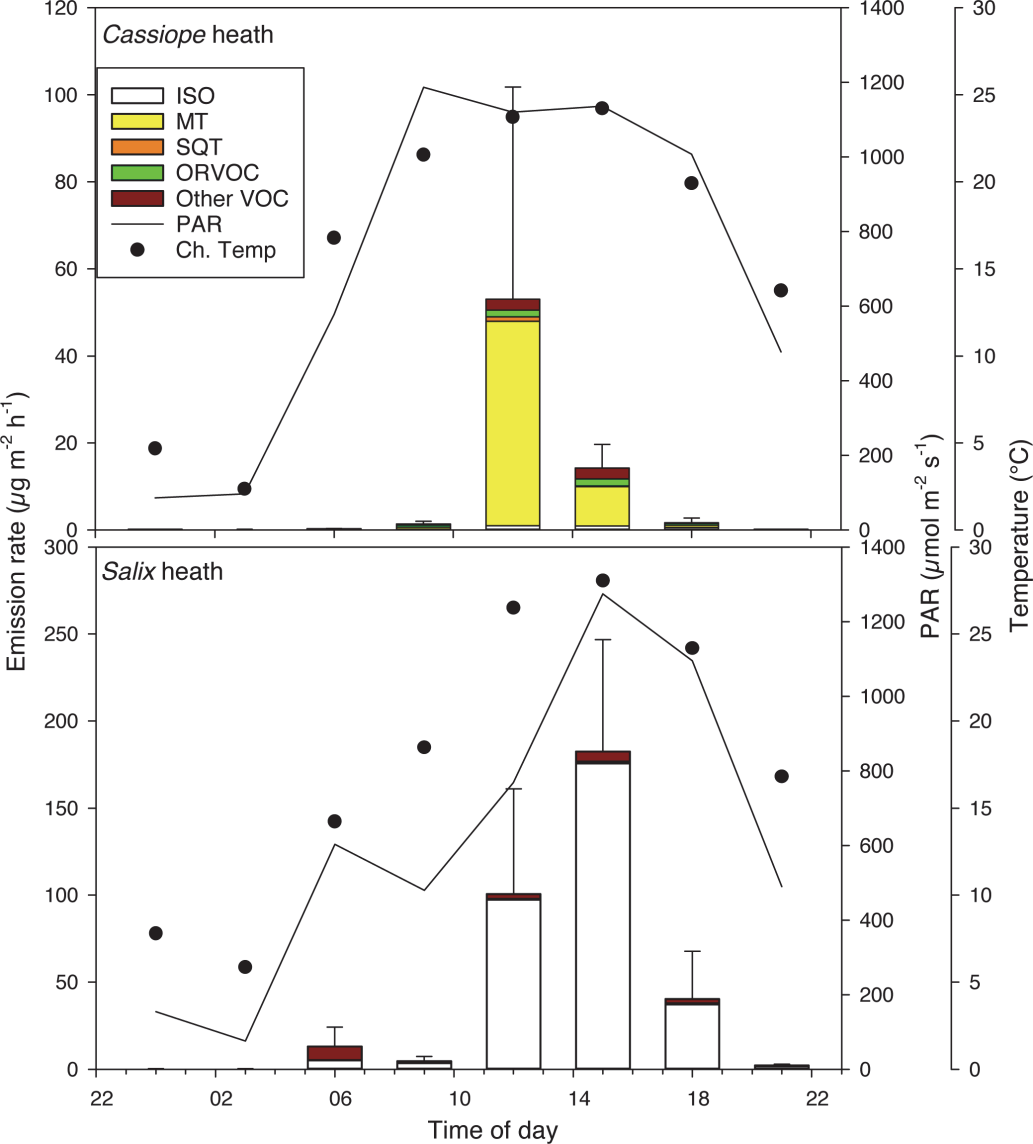
Diel emissions of biogenic volatile organic compounds (BVOCs) from a *Cassiope tetragona*-dominated heath and a *Salix arctica*-dominated heath in the High Arctic, Zackenberg valley. The stacked bars show mean emission rates (n = 4) of isoprene (ISO), monoterpenes (MT), sesquiterpenes (SQT), other reactive volative organic compounds (ORVOC) and other volative organic compounds (Other VOC) during a 24-hour period. The error bars represent standard error for total emissions. The incoming photosynthetically active radiation (PAR) and chamber temperature (Ch.Temp) during the measurements are shown.

At noon the total BVOC emission rate from the unmanipulated control plots in the *Cassiope*-heath was 53.0 ± 48.7 (mean ± SE) μg m^-2^h^-1^ (Fig 2 and S5 Table) and the rate for the *Salix*-heath was significantly higher, 100.6 ± 60.4 μg m^-2^h^-1^ (P<0.05, Fig 2 and S6 Table). The daily sum of total BVOC emissions during the 24-hour period tended to be lower for the *Cassiope*-heath than for the *Salix*-heath (P = 0.09; Table 1).

**Table 1:** Mean (SE) daily sums (n = 4) of BVOC emissions for high, low and subarctic sites.

Daily sums of BVOC emissions (μg m^-2^ d^-1^)							
Site	Treatment	Isoprene	MTs^a^	SQTs^b^	ORVOCs^c^	Other VOCs^d^	Total BVOCs^4^
**High Arctic**							
*Cassiope*-heath	Control	7.6 (4.6)	172 (132)	3.6 (3.0)	12.0 (4.0)	19.6 (6.9)	214.3 (144.9)
	Warming	46.1 (37.4)	94.2 (87.9)	12.0 (12.0)	21.8 (16.5)	29.5 (15.7)	203.6 (121.5)
*Salix*-heath	Control	961 (367)	0.3 (0.1)	<0.01	7.5 (2.8)	63.0 (18.5)	1032 (379.7)
	Warming	1918 (402)	1.3 (0.7)	<0.01	6.5 (4.3)	82.0 (26.8)	2008 (425.3)
**Low Arctic**							
*Betula*-heath		186 (35.4)	31.0 (14.3)	173.8 (69.3)	50.0 (13.3)	2919 (1477)	3357 (1447)
*Salix*-heath		1808 (808)	30.0 (21.4)	24.2 (18.7)	39.9 (19.2)	3029 (1643)	4931 (1790)
**Subarctic**							
July		736 (127)	1895 (139)	299 (11.8)	1995 (538)	5056 (1170)	10040 (1430)
August		2269 (619)	3584 (522)	51.7 (10.4)	1509 (395)	4164 (850)	11589 (1342)

^a^Monoterpenes (MTs)

^b^Sesquiterpenes (SQTs)

^c^other reactive volatile organic compounds (ORVOC; having a lifetime in the atmosphere less than 24 hours)

^d^other volatile organic compounds (Other VOCs; having a lifetime in the atmosphere longer than 24 hours)

^e^total biogenic volatile organic compounds (Total BVOCs).

Emissions from the *Cassiope*-heath peaked at noon and 90% of them were MTs (Fig 2 and S6 Table). The MT emissions were significantly lower from 21:00 to 09:00 compared to the 12:00 emissions (P<0.05). SQTs were emitted only at 12:00 and 15:00 from two plots at a maximum rate of 1.0 μg m^-2^h^-1^. The ORVOCs were emitted at < 2 μg m^-2^h^-1^ from 09:00 to 18:00 and at even lower rates at 21:00, midnight and 06:00 (P = 0.08). Emissions of other VOCs accounted for approximatively 1% of the total BVOC emissions and were significantly lower only at 21:00 compared to the noon emission rate (P<0.01). Isoprene was emitted in minor amounts during the daytime (S6 Table).

Isoprene, with a noon emission rate of 97.4 ± 61.2 μg m^-2^h^-1^, constituted 93% of the total emission from the *Salix*-heath (Table 1), with a maximum emission rate at 15:00 (175.7 ± 66.4 μg m^-2^h^-1^, S5 Table). The isoprene emission rate stayed high until 18:00 when it dropped and was significantly lower (P = 0.05) at all other time points compared to midday. The emissions of MTs and ORVOCs were low, with maximum rates of 0.04 ± 0.02 and 1.1 ± 0.8 μg m^-2^h^-1^, respectively, with no differences between time points during the 24-hour period (S6 Table). The noon emission rate of other VOCs was 2.8 ± 1.4 μg m^-2^h^-1^, and did not differ between time points (S6 Table). The *Salix*-heath did not release any SQTs.

The total BVOC and MT emissions from the *Cassiope*-heath correlated with PAR (P< 0.01, r^2^ = 0.29 for total BVOCs and 0.23 for MTs, n = 32) and chamber temperature (P< 0.01, r^2^ = 0.33 for total BVOCs and r^2^ = 0.28 for MTs, n = 32). There was a strong correlation between isoprene emission and PAR (P<0.001, r^2^ = 0.63, n = 32) and chamber temperature (P<0.001, r^2^ = 0.60, n = 32). The lowest PAR level was, between 21:00 and 06:00, on average 289 and 299 μmol m^-2^ s^-1^ for *Cassiope*- and *Salix*-heath, respectively (see Table 2 for 24-hour means).

**Table 2:** Mean (SE) abiotic parameters (n = 4) at the different sites during BVOC sampling.

Site	Treatment	Chamber Temp (°C)	Air Temp (°C)^a^	Soil moisture %	Soil Temp (°C) 2 cm	Soil Temp (°C) 5 cm	PAR^b^(μmol m^-2^ s^-1^)
**High Arctic**							
*Cassiope*-heath	Control	16.3 (1.5)	10.7 (0.6)	27.4 (1.7)	8.5 (0.3)	8.3 (0.2)	731 (79)
	Warming	16.7 (1.5)	10.7 (0.6)	25.6 (4.0)	9.6 (0.4)	9.2 (0.2)	597 (73)
*Salix*-heath	Control	17.8 (1.4)	14 (0.9)	22.5 (1.7)	9.1 (0.2)	8.6 (0.2)	618 (75)
	Warming	18.9 (1.5)	14 (0.9)	14.1 (1.7)	10.3 (0.3)	9.4 (0.2)	547 (68)
**Low Arctic**							
*Betula*-heath		12.3 (1.6)	7.0 (0.6)	66.1 (8.3)	7.8 (0.7)	6.7 (0.4)	809 (139)
*Salix*-heath		12.7 (1.5)	7.1 (0.7)	66.1 (8.3)	7.6 (0.6)	7.7 (1.1)	836 (141)
Light/Dark^c^	Control	21.9 (1.5)	14.9 (0.7)	43.0 (10.6)	8.6 (0.7)	6.1 (0.4)	1118 (170)
	Warming	28.6 (1.7)	14.5 (0.8)	26.8 (8.1)	9.4 (0.8)	5.4 (0.7)	1068 (212)
**Subarctic**							
July		14.4 (0.3)	n.a^d^	100	n.a^d^	n.a^d^	165 (22)
August		14.3 (0.6)	n.a^d^	100	n.a^d^	n.a^d^	245 (39)

^a^ The Air temperature was measured at 1.5 meters above the ground. There are no treatments included in the air temperature measurements.

^b^ PAR is the average measured PAR per campaign and 24-hour

^c^ Note that the values presented from the light/dark experiment (except PAR) are from measurements in both light and darkness.

^d^Data is not available (n.a)

#### Low Arctic

**Fig 3:**
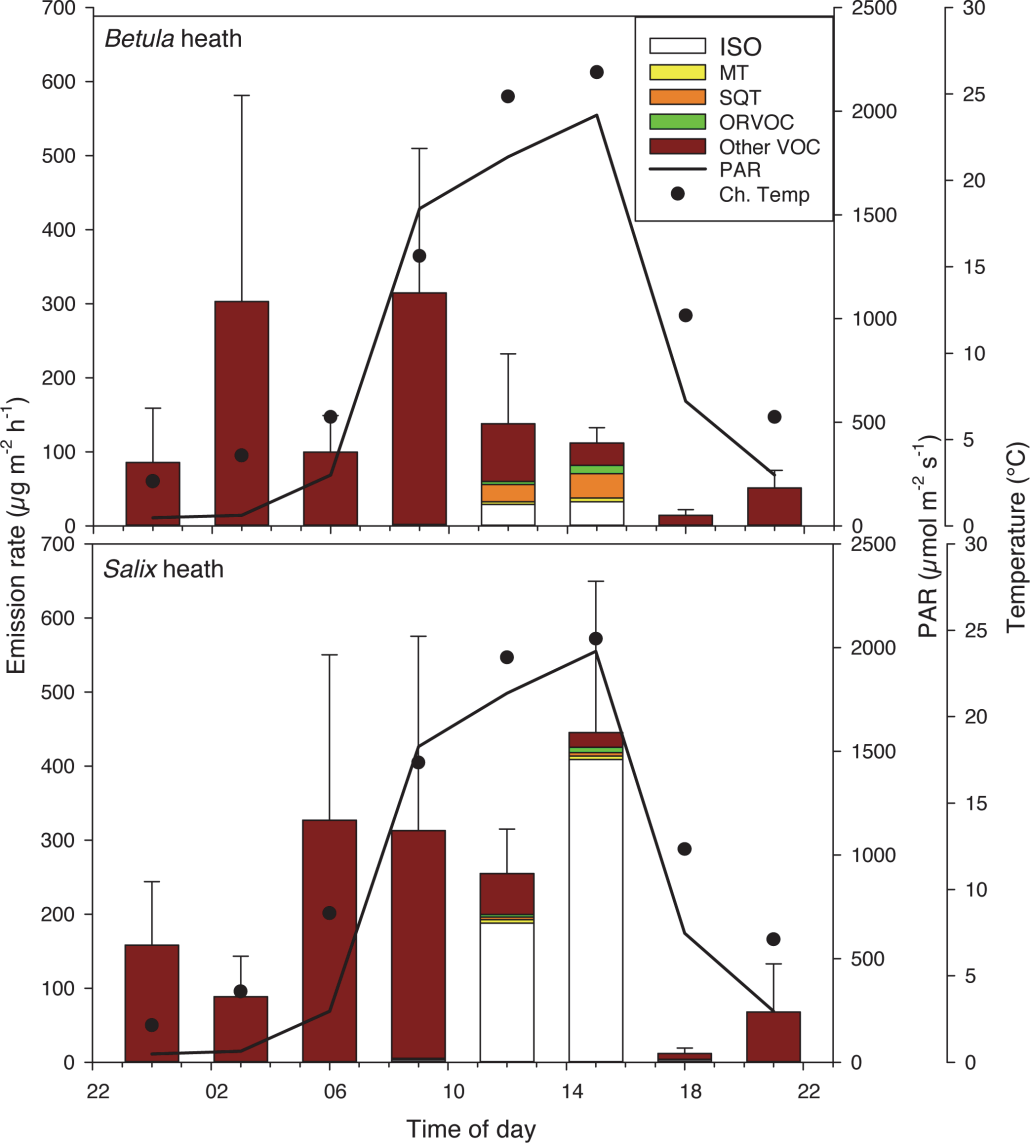
Diel emisisons of BVOCs from *Betula nana* and *Salix glauca*-dominated heaths in the Low Arctic, Disko Island. The stacked bars show mean emission rates (n = 4) of BVOCs during a 24-hour period. Abbreviations for the BVOC groups as in Fig 2. The error bars represent standard error for total emissions. The incoming photosynthetically active radiation (PAR) and chamber temperature (Ch.Temp) during the measurements are shown.

The total BVOC emission rates at 12:00 in the *Betula-* and *Salix*-heath in the low arctic site were 138.1 ± 94.3 and 254.7 ± 60.3 μg m^-2^h^-1^, respectively (Fig 3 and S7 Table). The daily sum of total BVOCs for *Betula-* and *Salix-* heaths did not differ from each other, and they were minimum 2 fold higher than the daily sum for the High Arctic (P<0.01; Table 1).

In the *Betula*-heath, isoprene was emitted only at 12:00 and 15:00 with emission rates of 29.2 ± 18.5 μg m^-2^h^-1^ and 32.6 ± 7.3 μg m^-2^h^-1^, respectively. The MT emission rates were low, varying between 0.3 and 5.2 μg m^-2^h^-1^, and only emitted from 09:00 to 21:00 with no differences between time points. Emission of SQTs occurred between 09:00 and 18:00, with an average of 23.2 ± 9.9 μg m^-2^h^-1^ at noon. The highest SQT emission rate of 32.8 ± 13.0 μg m^-2^h^-1^ at 15:00 did not differ from noon emissions but was higher than emissions at all other time points (P<0.001). The ORVOC emissions reached a maximum at 15:00 with an average of 11.1 ± 3.4 μg m^-2^h^-1^, which was significantly higher than at noon (P<0.01). The emission rate was low at all other time points and was under the detection limit from midnight to 09:00. The emission of other VOCs, with methyl-butane as the dominant compound, occurred throughout the 24-hour period, and was highest at 09:00, 312.6 ± 195.6 μg m^-2^h^-1^. There was a large variation in the emission of other VOCs and no significant differences were found between time points.

In the *Salix*-heath, the majority of emissions owed to isoprene, which was emitted from 09:00 to 18:00. The emission at 09:00 was 3.1 ± 2.0 μg m^-2^h^-1^, significantly lower compared to the rate at 12:00 (187.7 ± 78.9 μg m^-2^h^-1^; P<0.01; S7 Table). The emission stayed high until 15:00 at, 408.7 ± 190.0 μg m^-2^h^-1^, before dropping to 3.3 ± 2.0 μg m^-2^h^-1^ at 18:00 (P<0.01 compared to 12:00). MTs were emitted at three time points, 12:00, 15:00 and 21:00, with noon emission of 4.9 ± 4.3 μg m^-2^h^-1^. No significant differences between time points were found for SQTs, ORVOCs or other VOCs, which had noon emission rates of 3.6 ± 2.7, 3.3 ± 2.7 and 55.1 ± 37.5 μg m^-2^h^-1^, respectively. The daily sum of total BVOC emissions tended to be higher in the low arctic *Salix*-heath compared to the high arctic *Salix*-heath (P = 0.07; Table 1), which was due to the higher emission of other VOCs in the low arctic. The daily sum of isoprene emissions did not differ between the high- and low arctic *Salix*-heaths (P>0.1).

Isoprene, MT, SQT and ORVOC emissions correlated significantly with PAR (P<0.01, r^2^ = 0.34–0.58, n = 31) and temperature (P<0.01, r^2^ = 0.37–0.70, n = 31) for both vegetation communities. The lowest PAR level was between 21:00 and 06:00, at an average of 149.0 μmol m^-2^ s^-1^, (see Table 2 for 24-hour means).

#### Subarctic

In the subarctic ecosystem, where the moss *Warnstorfia exannulata* and the sedge *Eriophorum russeolum* were dominant species, the daily sum of total BVOCs in July was at a minimum two times higher than the daily sum for the high or low arctic ecosystem (P<0.01; Table 1). The daily sums of isoprene and MTs were significantly higher in August compared to July (P<0.05), but the emission of SQTs was higher in July compared to August (P<0.01; Table 1), and there were no significant differences between the daily sums of total BVOCs for the two samplings (P>0.1).

**Fig 4:**
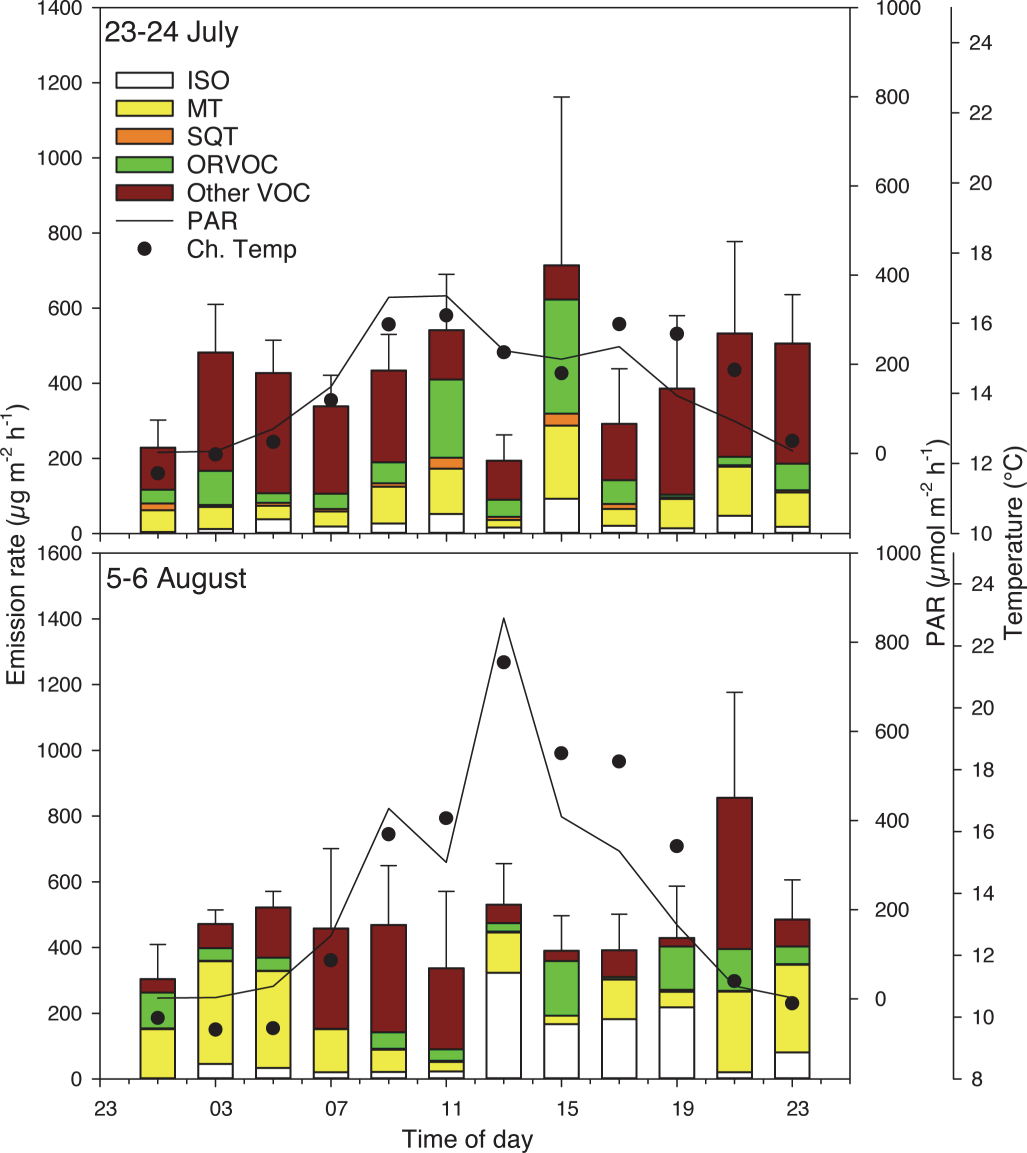
Diel emissions of biogenic volatile organic compounds (BVOCs) in the Subarctic, Northern Finland in July and August. The dominant plants in this site were the moss *Warnstorfia exannulata* and the sedge *Eriophorum russeolum*. The stacked bars show mean emission rates (n = 4) of BVOCs during two 24-hour periods. Abbreviations for the BVOC groups as in Fig 2. The error bars represent standard error for total emissions. The incoming photosynthetically active radiation (PAR) and chamber temperature (Ch.Temp) during the measurements are shown.

In July, the total BVOC emissions at 13:00 were less than one third, 188 ± 70 μg m^-2^h^-1^, of the highest emission found at 15:00, 692 ± 319 μg m^-2^h^-1^ (P = 0.07, Fig 4 and S8 Table). Isoprene emission was highest at 15:00, 93.1 ± 68.0 μg m^-2^h^-1^, and dropped to zero at 01:00 in all plots except for one that released 15.8 μg m^-2^h^-1^. Compared to midday, the MT emissions were significantly higher at 15:00, 19:00, 21:00, 23:00, 09:00 and 11:00 (P<0.01) and tended to increase at 03:00 and 05:00 (P = 0.07–0.08, S8 Table). The SQT emissions were 8.8 ± 5.3 μg m^-2^h^-1^ at 13:00 with no significant differences in emission rates during the 24 hours expect at 15:00 when the emissions increased three-fold to 31.9 ± 7.5 μg m^-2^h^-1^ (P<0.05). There were no significant differences in ORVOC and other VOC emissions between time points (S8 Table).

In August, there were no significant changes for total BVOC emissions between time points during the 24-hour period (Fig 4. and S9 Table). For isoprene, the highest rate was measured at 13:00, 323 ± 172 μg m^-2^h^-1^. Isoprene emission was under detection limit only at 01:00. No significant differences were found between time points for MTs, SQTs or other VOCs (P>0.1). The highest emission rate for ORVOCs was at 15:00, 165.8 ± 84.8 μg m^-2^h^-1^, which is significantly higher (P<0.05) than at 13.00, 25.5 ± 10.7 μg m^-2^h^-1^ (S9 Table).

Isoprene emissions in July did not correlate with either PAR or temperature (P>0.1). However, in August isoprene emission correlated with both PAR (P = 0.001, r^2^ = 0.2, n = 52) and temperature (P<0.001, r^2^ = 0.32, n = 52). The emission rates of other BVOCs did not correlate with PAR or temperature on either of the dates. The lowest PAR level was found between 21:00 and 07:00, at an average of 48 and 34 μmol m^-2^ s^-1^ in July and August respectively.

### Effect of warming on the diel variation of BVOC emissions

**Fig 5:**
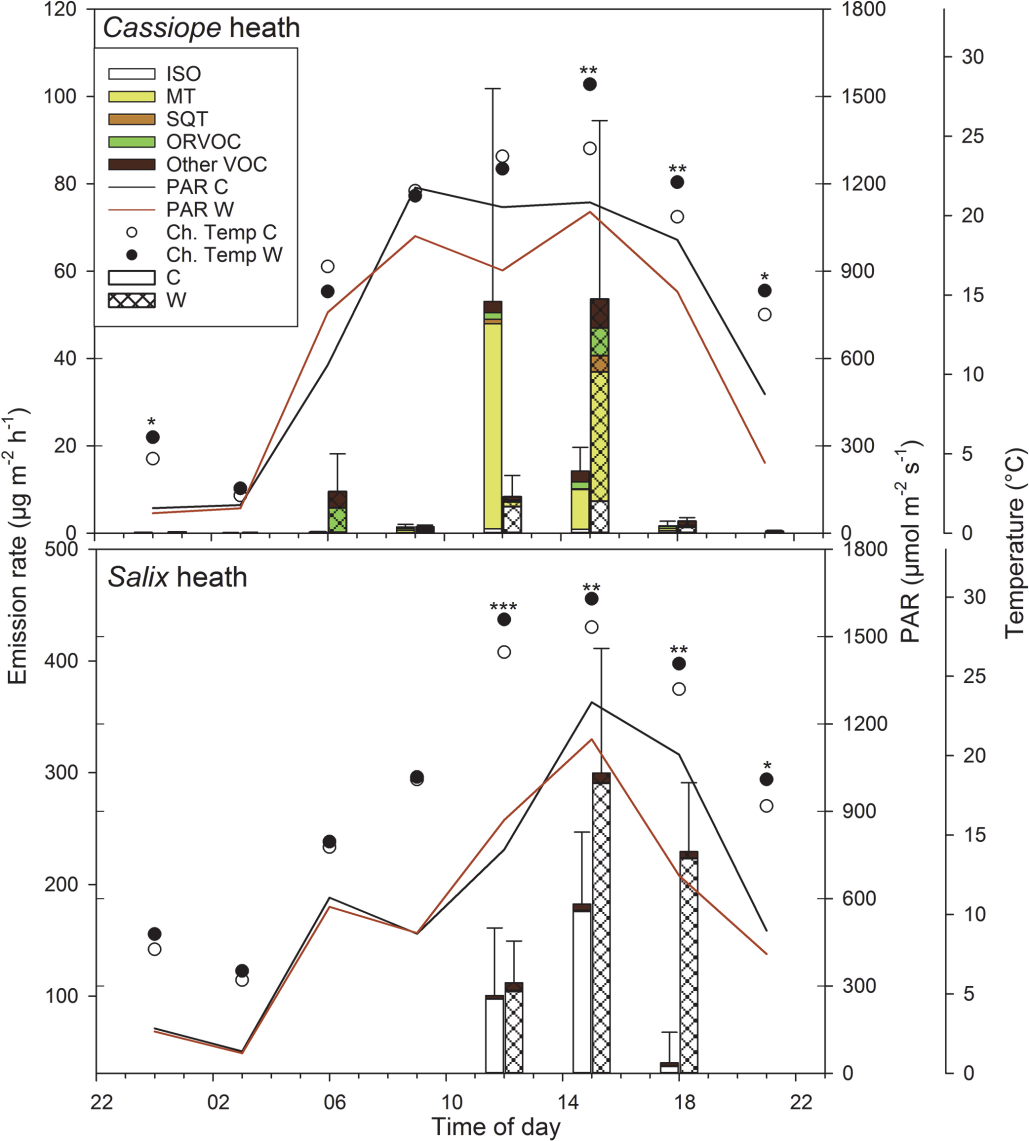
The effect of warming by open top chambers on the diel BVOC emissions in a high arcitc *Cassiope tetragona*-dominated heath and a high arctic *Salix arctica* **-**dominated heath. Mean BVOC emissions (n = 4) during a 24-hour period in control (C) and warmed (W) plots. Error bars represent standard error for the total emissions. Abbreviations for the BVOC groups as in Fig 2. The incoming photosynthetically active radiation (PAR) and chamber temperature (Ch.Temp) for the two treatments are shown. *(P<0.1), **(P<0.05) and ***(P<0.01) indicate significant differences in chamber temperature between treatments. Note different Y-axes scales for the different vegetation communities.

In the high arctic *Cassiope*-heath, only isoprene emission was significantly higher in the W compared to the C plots (P = 0.05, Fig 5 and S1 Table), and the contribution of isoprene to the total BVOC emission increased from 5% in the C to 21% in the W plots. No treatment effects were found for MTs, SQTs, ORVOCs or other VOCs. Testing emission rates separately for the time points revealed a trend that the total BVOC emission was increased by warming only at midnight (P = 0.08), but not at any other time (P>0.1). The daily sum of total BVOCs did not differ between C and W plots (P>0.1, Table 1).

In the *Salix*-heath, warming increased the 24-hour average BVOC emissions by 51% (P = 0.05, Fig 5 and S1 Table). The total BVOC emission tended to be increased by warming at 18:00 and 21:00 (P = 0.06 and P = 0.09). The overall isoprene emission was not significantly affected by warming, and of the different time points only at 18:00; isoprene emission was 83% higher in W than C plots (P<0.05, Fig 5). The MT emissions were increased by 18% in the W compared to the C plots at 12:00 (P = 0.01). The MTs emitted from the *Salix*-heath only contained 1,8-cineole in the C plots and 1,8-cineole and β-ocimene in the W plots, both 1,8-cineole and β-ocimene emissions correlated with chamber temperature (P<0.01 and <0.05 and r^2^ = 0.3 and 0.2 respectively). There was no difference in the daily sum of total BVOCs between C and W plots (P>0.1, Table 1).

In the *Cassiope*-heath measurement, the chamber temperature did not differ between the treatments averaged over the 24 hours (P>0.1). It was significantly higher in the W compared to the C plots between 15:00 and 00:00 (P<0.05) but no differences were found between 03:00 and 12:00 (Fig 5). In contrast, the 24-hour average chamber temperature in the *Salix*-heath was significantly warmer by 1.1°C in the W than in the C plots (P<0.001) and this difference was significant between 12:00 and 21:00 (Fig 5). No difference was found at 00:00 and 09:00. Warming increased the soil temperature at 2 cm depth by 1.1 and 1.2°C and at 5 cm depth by 0.8 and 0.9°C for *Cassiope*-heath and *Salix*-heath, respectively (P<0.05, Table 2). There was a positive correlation between soil and chamber temperature, averaged for both C and W plots (P<0.001, r^2^ = 0.23 and r^2^ = 0.24 for *Cassiope-* and *Salix*-heath respectively). The soil moisture did not differ between the treatments in the *Cassiope*-heath (P>0.1), but it was significantly reduced by warming in the *Salix*-heath (P<0.05, Table 2). The OTCs used for warming did not affect the incoming PAR (P>0.1). The total plant coverage in the *Cassiope*-heath did not differ between treatments while an 18% higher plant cover was found in the W compared to the C plots in the *Salix*-heath (P<0.05); the additional coverage consisted of the species *S*. *arctica*, and *Dryas octopetala* (S1 Table).

### Effects and interactions between light manipulation and warming on BVOC emissions

**Fig 6:**
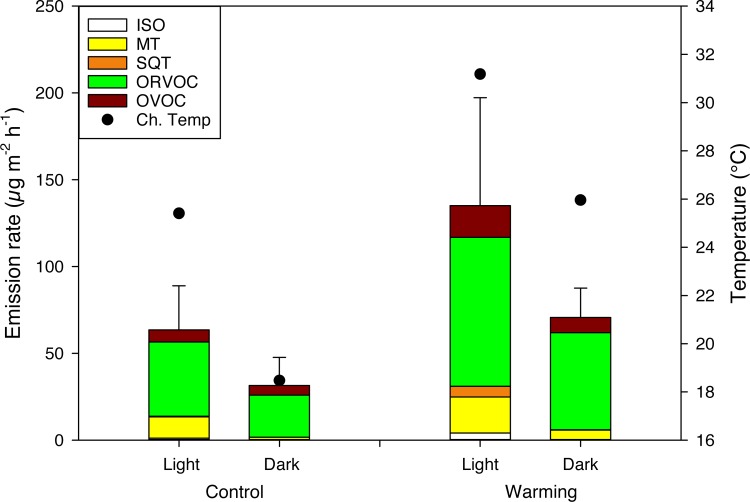
The effect of darkening on BVOC emissions in a low arctic heath under ambient and warmed conditions. Measurements were conducted with a transparent chamber (light) and with the chamber darkened with a black cloth (dark) in two field treatments: control and warming. Error bars represent standard error for total emissions (n = 4). Abbreviations for the BVOC groups as in Fig 2. Mean chamber temperatures (Ch.Temp) are shown.

In the low arctic light/dark measurements, the temperature inside the measurement chambers was reduced by 6.2°C due to darkening (P<0.05 averaged for both C and W plots). The total BVOC emissions, averaged for both C and W plots, were 48% higher in light compared to darkness (S10 Table). However, this difference was not statistically significant when chamber temperature, which had a significant positive effect on the emissions (P = 0.01), was used as a covariate (Fig 6). Also the increased number of compounds emitted in light than in darkness (average for both C and W plots 15 ± 2.5 compared to 10 ± 1) was due to the higher temperature (P<0.05).

There was no isoprene emission from C or W plots during dark measurements, and the emission measured in light was not affected by chamber temperature (P>0.1). MT emission was lower in dark than light due to a decreased chamber temperature (P<0.05 average for both C and W plots, Fig 6). Of the individual MTs, α-thujene, α-fenchene, α-phellandrene, β-pinene, 3-carene, α-terpinene and fenchol were only emitted in light, where light emission of α-fenchene, 3-carene and α-terpinene correlated with chamber temperature (P = 0.03, 0.02 and 0.06 and r^2^ = 0.57, 0.81 and 0.47, respectively). The individual MTs, α–pinene, camphene and d-limonene were not affected by the light manipulation (P>0.1). The SQT emissions were in general higher in light, because of the higher chamber temperature (P>0.05 for light/dark factor and P<0.05 for chamber temperature), and there was an interaction between the light manipulation and field treatments (P<0.05). A separate test revealed that the SQT emissions in C plots were significantly higher in the light measurements, due to the higher chamber temperature (P<0.01), but the light treatment itself was not a controlling factor for the emissions (P>0.1). There was a trend that chamber temperature and light increased the emissions of SQTs in the W plots (P = 0.07 and P = 0.1, respectively). Chamber temperature had no effect on SQT emissions in the dark measurements (P>0.1) but was a controlling factor in the light measurements (P<0.05). The ORVOC emission rates were 38% higher in light than in dark, however, this was due to increased temperature (P>0.05 for light/dark factor and P<0.05 for chamber temperature). Emission of other VOCs were unaffected by the light manipulation and chamber temperature (P>0.1).

The number of different compounds emitted, averaged for both light and dark measurements, was significantly higher in the W than in the C plots (P<0.05), 16 ± 2 compared to 10 ± 2. The emissions of other VOCs and SQTs were significantly increased by warming (P<0.05).

The chamber temperature, averaged for light and dark measurements, was 6.7°C higher in the W than in the C plots (P<0.05, Table 2). The soil temperatures at 2 and 5 cm depths did not differ between the W and C plots or light manipulations (P>0.1; Table 2). The incoming PAR during the measurements in light did not differ between the field treatments (P>0.1). The total plant coverage did not differ between the treatments (P>0.1).

## Discussion

### Variation in emission over 24 hours

This study shows that BVOCs are emitted from arctic ecosystems during both day- and night-time, which is in agreement with our hypotheses. The night-time emissions were in general significant, albeit lower than those during the day, and thus they should not be overlooked when considering the total emissions. In particular, the relatively stable emissions in the subarctic site throughout the 24 hours should be noted (Fig 4).

In the high arctic heaths, BVOC emissions correlated with PAR and temperature and were very low during night-time (Fig 2). However, the emissions dropped substantially by 21:00, even though the PAR was still relatively high (480 μmol m^-2^ s^-1^) and the temperature was well above the 10°C suggested as a minimum for significant BVOC emissions from these ecosystems [38]. It seems likely that the emissions of some BVOCs follow a circadian cycle not corresponding to the available light levels, as has been shown for the emission of BVOCs from many plants [7,39,40]. This conclusion is also supported by an earlier finding demonstrating that carbon assimilation rates for *Salix pulchra* Cham. and *C*. *tetragona* from Tussock tundra in Alaska (68°37’ N) follow a circadian cycle [38]. Patankar et al. [38] reported peaks in carbon assimilation rates at 12:00 for *Salix* and between 8:00 and 12:00 for *Cassiope* and very low rates during night-time despite PAR levels above saturation. Thus, the plants did not take advantage of the available light during evenings [38].

In the low arctic heath, no clear diel patterns for the total emissions were apparent (Fig 3). Isoprene emission was highest during the day and correlated with light and temperature, which is in line with the general understanding of BVOC emission behavior [7,11]. The emissions of other VOCs were stable throughout the 24-hour period and they were the only compound group emitted during night-time. Other VOCs may be released by the soil and the emissions of e.g. 2-methyl-butane and methoxy-phenyl-oxime may therefore be dependent on soil characteristics such as microbial biomass and activity, nutrient availability, amount and quality of litter, and soil moisture [41,42] more so than on light and/or air temperature. Nevertheless, soil emissions are often 1–2 orders of magnitude lower than the emission from aboveground plant parts [43], and thus, it is not likely that all other VOC emissions derive from the soil. The few studies on soil emissions from northern ecosystems show contrasting results [15,42] suggesting possibly higher contribution of soil emission for arctic ecosystems with low leaf area index and large carbon stocks in the soil. However, in general the importance of these emissions to the ecosystem flux is not known [43].

In the subarctic site, many non-oxygenated terpenes, for example isoprene, d-limonene, 3-carene, α-pinene and β-caryophyllene, showed no clear differences between day- and night-time emissions (Fig 4). Isoprene emission during the night, when the PAR was zero, was a surprise result as this BVOC is produced light-dependently [7]. Isoprene emitted during the night in darkness may derive from temporary storage pools rather than *de novo* synthesis. The presence of temporary storage pools for isoprene has been suggested by Funk et al. [44] and Wiberley et al. [45] who studied emission of isoprene from *Populus* species. Wiberley et al. [45] reported isoprene emission after three days of darkness from Poplar trees, indicating that isoprene originated from storage pools. Night-time emission of MTs, such as α-pinene, β-pinene, 3-carene and limonene, has earlier been reported [24,46]. The night-time emissions of these MTs are likely to originate from temporary storage pools, being independent of light or stomatal conductance (for details on stomatal conductance in controlling BVOC emissions see Harley [47]), because the dominant plant species, *W*. *exannulata* and *E*. *russeolum*, in the subarctic site probably lack specialized storage structures. However, isoprene [48] and MTs [41] emitted during night-time may also derive from the soil, which cannot be separated from whole ecosystem flux in the present study.

Afternoon peaks in SQT emissions were found in the low arctic site as well as in the subarctic site in July. Diel variation in SQT emissions has earlier been reported, with peaks in the morning for Black Sage (*Salvia mellifera*) in California [49] and at noon for various *Citrus* varieties in Spain [50]. SQTs have also been shown to be emitted in dark and at night [50–53]. Thus, there are differences in emission patterns of SQTs between different species and habitats, and it is unclear what factors control their diel variation [53].

The emission profiles differed between day- and night-time, at all three sites. Also, the emission rates differed between sites. The highest emission rates and daily sum of total BVOC emissions were found at the subarctic site and the lowest in the high arctic site, which suggests that the emission rates decrease towards higher latitudes. However, even the high arctic emission rates during daytime are in a range that cannot be ignored, as also shown by Schollert et al. [6].]. In fact, the isoprene emission from the high arctic was as high as the isoprene emission found in the low arctic and in the subarctic peatland in the present study, as well as earlier reported for a subarctic wet heath [30]. The isoprene emission from the high arctic *Cassiope*-heath was comparable to the rates for a similar vegetation community in Schollert et al. [6]. However, the isoprene emission measured for the high arctic *Salix*-heath was much higher than in Schollert et al. [6], (176 ± 66 compared to 7.9 ± 2.1 μg m^-2^h^-1^). The dissimilarity in reported emission rates could be explained by different environmental conditions or a seasonal effect as Schollert et al. [6] measured in August while the measurements in the present study were conducted in July. The differences in BVOC emission rates between sites could be explained by differences in plant species composition, plant biomass, soil properties and activity of microorganisms, or environmental conditions. The higher daily sum of isoprene measured in August compared to July in the subarctic ecosystem is probably due to the higher PAR and chamber temperature driving high daytime emissions during the August measurement. In contrast, the higher MT emissions in August compared to July could not be explained by the light levels or chamber temperature, since the higher emissions occurred during night-time and did not correlate with PAR or temperature. The difference might be due to a seasonal effect or a build-up of temporary storage pools during the warm day that was released during night-time.

In general, the night-time emissions during the midnight sun period were lower than expected. The low emissions could be partly explained by the low light levels during late evenings and night, which was due to the sun being obstructed by mountains and terrain. Thus, despite the location north of the Arctic Circle (66°N) and the measurements within the midnight sun period, the PAR was low during the night. Alternatively, changes in deposition rates of BVOCs may also explain the low net emissions of BVOCs during night-time [54], but deposition processes could not be studied with the methodology used in the present study.

### Effect of warming on BVOC emissions

The OTC warming in the high arctic heath increased the temperature during the measurements both during the day and night, which is in contrast to our hypothesis and earlier data [31] suggesting that the effect of the OTC warming is driven by PAR. However, the most significant difference in temperature was found during the day (Fig 5). The significant warming effect during the night when PAR was very low may be explained by the warmed soil inside the OTCs. Soil, which has low albedo [55] and higher heat capacity than the surrounding air [56], warms up during the day and acts as a temperature buffer during the night.

The total BVOC emissions were significantly increased by warming in the high arctic *Salix*-heath. It was expected that the increased emission would be due to a higher emission of isoprene, since it is the most emitted single compound from the *Salix*-heath and isoprene emission from a tundra heath has been reported to increase with increased temperature [28]. However, isoprene emission significantly increased under warming only at 18:00 while the total amount of emission over the 24-hour period was unaffected. The only compound group for which the 24-hour emissions were significantly increased by warming was MTs, of which only β-ocimene was significantly affected. The lipophilic β-ocimene is able to diffuse through the cell membrane [47], and the emissions correlated positively with chamber temperature. It is therefore likely, that this increase was due to a higher diffusion rate from the specialized storage structures on the leaves [57]. The lower soil moisture in W plots may have induced stomatal closure, which can reduce the production and emission of BVOCs coming directly from *de novo* synthesis or temporary storage pools inside the leaves. Thus, the emissions may increase more significantly in a future warmer and wetter [58] climate, than what was shown here.

The increase in BVOC emission rates can be due to higher temperature, leading to higher production and diffusion rate. Moreover, changes in the amount or composition of vegetation could affect emissions. The plant cover in the high arctic *Salix*-heath was increased by 18% in warmed plots. The increased plant cover, and consequent higher BVOC production may have contributed to the higher emission rates. In the *Cassiope*-heath, isoprene emission was higher over the 24-hour period under warming. The vegetation cover in the control and warmed plots were not significantly different and we suggest that the increased isoprene emission was due to stimulated *de novo* synthesis due to higher temperature.

The 6.7°C warming caused by the OTCs in the low arctic heath resulted in increased emissions of SQTs and other VOCs. The total BVOC emissions increased under warming in the high arctic *Salix*-heath, but, no clear treatment effects on the emission of any group or total BVOCs from the high arctic *Cassiope*-heath due to large variation in the data. We suggest that future studies should focus on the processes that affect the emissions from different vegetation communities under changing soil moisture and temperature conditions, using, for example, carbon isotope labeling. If the plant biomass of these areas increases as suggested by Hudson et al. [59] we can expect that a future warmer climate will increase the BVOC emissions from the Arctic [60].

### Effect of light and dark on BVOC emissions

The emission rates of most compounds and the number of different compounds emitted were clearly lower in dark than in light during the day (Fig 6), showing an immediate effect of light conditions. However, the correlation between temperature and light makes it difficult to separate the driving factors for emissions. Isoprene, α-fenchene, α-phellandrene, 3-carene, α-terpinene, α-selinene and fenchol were only emitted under ambient light, and the emissions of oxygenated MTs were in general very low in dark measurements, suggesting that the emissions of these compounds are dependent on the rate of *de novo* synthesis, stomatal conductance or chamber temperature, or a combination thereof [47]. Our finding is in agreement with an earlier report showing that the emission rates of many MTs decreased when a measurement cuvette was darkened during sampling from branches of *Betula pubescens* in southern Finland [30]. Thus, it is evident that emissions of some compounds are directly dependent on the light conditions or indirectly dependent on stomatal conductance. Emissions during darkness may result from diffusion from the temporary storage pools and some compounds may have been released in the beginning of the sampling period due to a slow response in stomatal conductance to darkening that started only 10 minutes prior to sampling. Also, we cannot rule out that the lower temperature during darkening had an effect on the diffusion rate and production of BVOCs. Thus, studies under controlled conditions are needed to further understand the light responses.

## Conclusion

BVOC emissions from arctic and subarctic ecosystems are of importance, even during night-time, and should be considered in global models. The emissions during the night in the low and subarctic sites were in the same range as during the day, which warns against overlooking the night period when considering emissions from arctic regions.

Large differences in diel variation of BVOC emissions were found between sites examined in this study. This variation may be explained by different dominant plant species, seasonal effects as well as differences in environmental parameters, such as temperature, light and soil moisture. These results highlight the importance of reporting what time of the day measurements are conducted, as the BVOC profiles change over the 24-hour period. This point is also important when studying treatment effects on BVOC emissions since the effect seems to differ between times of the day. We suggest that future studies focus on factors regulating the diel variation of the emissions in the arctic region, which would improve the accuracy of the BVOC emission estimates for the Arctic.

## Supporting Information

S1 Table The mean coverage (SE) of plant species in the control and warmed plots of a *Cassiope tetragona*- and a *Salix arctica-*dominated heaths, in the high arctic Zackenberg Valley, mid-July 2013. Vegetation coverage was analyzed using the point-intercept method (n = 4).
(PDF)

S2 Table The mean coverage (SE) of plant species in the *Betula-* and *Salix-* dominated heath, on the low arctic Disko island, mid-June 2013. Vegetation coverage was analyzed using the point-intercept method (n = 4).
(PDF)

S3 Table The mean coverage (SE) of plant species in in the subarctic site, Sodankylä Northern Finland, mid-July 2008. Vegetation coverage was analyzed using the point-intercept method (n = 4).
(PDF)

S4 Table The mean coverage of plant species (SE) in the control and warmed plots in the light/dark experiment (n = 4) on the low arctic Disko Island, mid-July 2013. Vegetation coverage was analyzed using the point-intercept method.
(PDF)

S5 Table Mean (SE) biogenic volatile organic compound (BVOC) emissions from a high arctic *Cassiope*-dominated heath in control and warming treatments (n = 4) during a 24-hour period the 24–25 July. (PDF)

S6 Table Mean (SE) biogenic volatile organic compound (BVOC) emissions from a high arctic *Salix*-dominated heath in control and warming treatments (n = 4) during a 24-hour period the 25–26 July. (PDF)

S7 Table Mean (SE) biogenic volatile organic compound (BVOC) emissions from one *Betula-* and one *Salix*-dominated heath (n = 4) in the Low Arctic during a 24-hour period the 18–19 June 2013. (PDF)

S8 Table Mean (SE) biogenic volatile organic compound (BVOC) emissions from a subarctic peatland (n = 4) during a 24-hour period the 23–24 of July 2008. (PDF)

S9 Table Mean (SE) biogenic volatile organic compound (BVOC) emissions from a subarctic peatland (n = 4) during a 24-hour period the 5–6 of August 2008. (PDF)

S10 Table Mean (SE) biogenic volatile organic compound (BVOC) emissions under light and dark conditions in control (C) and warming (W) treatment (n = 4) from a low arctic heath. (PDF)
