# Supplementary material for: Diel Variation of Biogenic Volatile Organic Compound Emissions- A field Study in the Sub, Low and High Arctic on the Effect of Temperature and Light
Source: PLoS One. 2015 Apr 21;10(4):e0123610. doi: 10.1371/journal.pone.0123610 (PMC4405581; doi:10.1371/journal.pone.0123610)
Supplement: S1 Table — Vegetation coverage was analyzed using the point-intercept method (n = 4). (PDF) [file pone.0123610.s001.pdf]

Table S1. The mean coverage (SE) of plant species in the control and warmed plots of a *Cassiope tetragona*- and a *Salix arctica*-dominated heaths, in the high arctic Zackenberg Valley, mid-July 2013. Vegetation coverage was analyzed using the point-intercept method (n=4).

Vegetation type	Plant species	Percentage cover			
		Cassiope-dominated		*Salix*-dominated	
		**Control**	**Warming**	**Control**	**Warming**
Deciduous shrubs					
	Salix arctica	3.6 (2.9)	2.6 (1.5)	31.6 (4.8)	51.5 (2.9)
	Vaccinium uliginosum	0	0	26.5 (2.2)	25.0 (9.9)
Evergreen shrubs					
	Cassiope tetragona, green	12.2 (7.8)	12.2 (0.8)	0	0
	Cassiope tetragona, brown	14.8 (5.2)	9.7 (2.7)	0	0
	Cassiope tetragona, grey	46.9 (8.0)	35.2 (3.9)	0	0
	Dryas octopetala			6.6 (6.0)	12.8 (12.8)
Gramminoids					
	Arctagrostis latifolia	1.5 (1.5)	0	2.6 (2.6)	5.1 (3.9)
	Carex bigelowii	0	0	3.1 (3.1)	2.0 (2.0)
	Hierochloe alpina	0	0	4.6 (4.6)	0
	Luzula arctica	2.6 (2.6)	0	0	0
	Poa arctica	0	5.6 (5.6)	13.3 (4.6)	0.5 (0.5)
Forbs					
	Bistorta vivipara	1.5 (1.5)	3.6 (1.3)	0	2.0 (1.2)
Mosses					
	Mosses	21.9 (4.3)	15.8 (1.7)	23.5 (4.5)	27.0 (8.1)
Lichens					
	Cetraria islandica	4.1 (2,9)	6.6 (2.7)	0	0
	Stereocaulon sp.	2.6 (2.6)	0	0	0
	Other lichens	2.6 (1.5)	4.6 (2.1)	3.6 (2.4)	6.6 (3.5)
Litter					
	Litter	23.5 (5.6)	15.3 (1.0)	31.6 (5.0)	41.8 (4.1)
Soil/crust					
	Bare soil	8.2 (4.2)	14.8 (3.9)	4.6 (2.9)	4.6 (2.1)
	Cryptogamic crust	2.6 (1.5)	3.6 (3.6)	6.1 (1.7)	3.6 (2.4)
