# Supplementary material for: Diel Variation of Biogenic Volatile Organic Compound Emissions- A field Study in the Sub, Low and High Arctic on the Effect of Temperature and Light
Source: PLoS One. 2015 Apr 21;10(4):e0123610. doi: 10.1371/journal.pone.0123610 (PMC4405581; doi:10.1371/journal.pone.0123610)
Supplement: S2 Table — Vegetation coverage was analyzed using the point-intercept method (n = 4). (PDF) [file pone.0123610.s002.pdf]

Table S2. The mean coverage (SE) of plant species in the *Betula*- and *Salix*- dominated heath, on the low arctic Disko island, mid-June 2013.

Vegetation coverage was analyzed using the point-intercept method (n=4).

**Vegetation type**	**Plant species**	**Percentage cover**	
		***Betula*-dominated**	***Salix*-dominated**
Deciduous shrubs			
	Betula nana	52.6 (4.2)	0
	Salix glauca	4.1 (-)[a]	41.8 (4.3)
	Vaccinium uliginosum	21.8 (3.6)	23.0 (5.0)
Evergreen shrubs			
	Rhododendron lapponica	6.1 (-)[a]	0
Graminoids			
	Carex bigelowii	3.1 (1.0)	0
	Carex spp.	30.6 (5.4)	15.8 (5.7)
Forbs			
	Bistorta vivipara	6.6 (3.4)	8.2 (2.5)
	Pedicularis flammea	6.1 (1.2)	4.1 (2.0)
Mosses			
	Mosses	30.6 (8.0)	37.2 (10.4)
Lichens			
	Cetraria islandica	5.1 (1.0)	2.0 (-)[a]
	Cladonia rangiferina	4.1 (-)[a]	0
Litter			
	Litter	46.4 (7.0)	56.1 (8.9)
	Standing litter	5.1 (1.0)	16.3 (-)[a]

[a] (-) the species was found in only one plot.
