# Supplementary material for: Diel Variation of Biogenic Volatile Organic Compound Emissions- A field Study in the Sub, Low and High Arctic on the Effect of Temperature and Light
Source: PLoS One. 2015 Apr 21;10(4):e0123610. doi: 10.1371/journal.pone.0123610 (PMC4405581; doi:10.1371/journal.pone.0123610)
Supplement: S3 Table — Vegetation coverage was analyzed using the point-intercept method (n = 4). (PDF) [file pone.0123610.s003.pdf]

Table S3. The mean coverage (SE) of plant species in in the subarctic site, Sodankylä Northern Finland, mid-July 2008. Vegetation coverage was analyzed using the point-intercept method (n=4).

Vegetation type	Plant species	Percentage cover
Evergreen shrubs		
	Vaccinium oxycoccos	5.8 (-)[a]
	Andromeda polifolia	7.4 (-)[a]
Graminoids		
	Carex limosa	6.8 (2.2)
	Eriophorum russeolum	68.2 (6.4)
	Scheuchzeria palustris	3.3 (4.3)
Forbs		
	Menyanthes trifoliata	2.5 (-)[a]
Moss		
	Warnstorfia exannulata	55.4 (24.0)
Dead material		
	Mud buttom	35.0 (8.9)

[a] (-) the species was found in only one plot.
