# Supplementary material for: Diel Variation of Biogenic Volatile Organic Compound Emissions- A field Study in the Sub, Low and High Arctic on the Effect of Temperature and Light
Source: PLoS One. 2015 Apr 21;10(4):e0123610. doi: 10.1371/journal.pone.0123610 (PMC4405581; doi:10.1371/journal.pone.0123610)
Supplement: S4 Table — Vegetation coverage was analyzed using the point-intercept method. (PDF) [file pone.0123610.s004.pdf]

Table S4. The mean coverage of plant species (SE) in the control and warmed plots in the light/dark experiment (n=4) on the low arctic Disko Island, mid-July 2013. Vegetation coverage was analyzed using the point-intercept method.

**Vegetation type**	**Plant species**	**Percentage cover**	
		**Control**	**Warming**
Deciduous shrubs			
	Betula nana	0	8.2 (-)[a]
	Vaccinium uliginosum	12.2 (4.2)	8.2 (3.1)
Evergreen shrubs			
	Empetrum nigrum	23.8 (5.5)	8.2 (-)[a]
	Rhododendron tomentosum	2.0 (-)[a]	6.1 (-)[a]
	Cassiope tetragona, green	12.2 (-)[a]	15.3 (6.6)
	Cassiope tetragona, brown	8.2 (-)[a]	7.7 (2.3)
	Cassiope tetragona, grey	16.3 (6.1)	32.7 (6.3)
	Pyrola grandiflora	6.1 (1.2)	5.1 (3.1)
Forbs			
	Bistorta vivipara	2.0 (0.0)	0
	Tofieldia pusilla	2.0 (-)[a]	0
Mosses			
	Mosses	4.1 (2.0)	17.9 (3.4)
Lichens			
	Cetraria islandica	16.3 (8.3)	8.2 (0.0)
	Cetraria nivalis	6.1 (-)[a]	18.4 (2.0)
	Cladonia rangiferina	2.0 (-)[a]	0
	Stereocaulon paschale	4.1 (0.0)	2.0 (-)[a]
	Cetrariella delisei	0	4.1 (-)[a]
Litter			
	Litter	18.4 (6.1)	19.9 (7)
	Standing litter	24.5 (9.0)	10.9 (4.8)
Cryptogamic crust			
	Cryptogamic crust	5.1 (1.0)	5.4 (1.8)

[a](-) the species was found in only one plot.
