# Supplementary material for: Diel Variation of Biogenic Volatile Organic Compound Emissions- A field Study in the Sub, Low and High Arctic on the Effect of Temperature and Light
Source: PLoS One. 2015 Apr 21;10(4):e0123610. doi: 10.1371/journal.pone.0123610 (PMC4405581; doi:10.1371/journal.pone.0123610)
Supplement: S5 Table — (PDF) [file pone.0123610.s005.pdf]

Table S5. Mean (SE) biogenic volatile organic compound (BVOC) emissions from a high arctic *Cassiope*-dominated heath in control and warming treatments (n=4) during a 24-hour period the 24-25 July.

Emission (μg m^{-2} h^{-1})	Control								Warming							
Time	00:00	03:00	06:00	09:00	12:00	15:00	18:00	21:00	00:00	03:00	06:00	09:00	12:00	15:00	18:00	21:00
Isoprene	<0.01	<0.01	<0.01	<0.01	1.01 (1.01)	0.93 (0.93)	0.59 (0.59)	<0.01	<0.01	<0.01	<0.01	0.56 (0.34)	6.13 (5.34)	7.36 (6.02)	1.33 (1.06)	<0.01
Monoterpenes																
α-thujene	<0.01	<0.01	<0.01	<0.01	0.57 (0.57)	1.06 (1.06)	<0.01	<0.01	<0.01	<0.01	<0.01	<0.01	<0.01	<0.01	<0.01	<0.01
α-pinene	<0.01	<0.01	<0.01	0.11 (0.07)	0.19 (0.14)	0.52 (0.52)	0.07 (0.07)	<0.01	<0.01	<0.01	0.10 (0.06)	0.11 (0.11)	0.07 (0.07)	0.15 (0.15)	0.11 (0.07)	<0.01
α-fenchene	<0.01	<0.01	<0.01	<0.01	<0.01	<0.01	<0.01	<0.01	<0.01	<0.01	<0.01	<0.01	<0.01	0.15 (0.08)	<0.01	<0.01
Camphene	<0.01	<0.01	<0.01	0.02 (0.02)	0.59 (0.47)	0.22 (0.17)	<0.01	<0.01	<0.01	<0.01	<0.01	<0.01	0.07 (0.07)	0.08 (0.08)	<0.01	<0.01
β-pinene	<0.01	<0.01	<0.01	<0.01	0.14 (0.09)	0.23 (0.15)	0.03 (0.03)	<0.01	<0.01	<0.01	<0.01	<0.01	<0.01	0.03 (0.03)	<0.01	<0.01
β-myrcene	<0.01	<0.01	<0.01	<0.01	<0.01	<0.01	<0.01	<0.01	<0.01	<0.01	<0.01	<0.01	<0.01	1.61 (1.61)	<0.01	<0.01
α-phellandrene	<0.01	<0.01	<0.01	<0.01	1.18 (1.14)	0.44 (0.35)	<0.01	<0.01	<0.01	<0.01	<0.01	<0.01	0.06 (0.06)	1.02 (1.02)	<0.01	<0.01
α-terpinene	<0.01	<0.01	<0.01	<0.01	6.59 (6.44)	<0.01	<0.01	<0.01	<0.01	<0.01	<0.01	<0.01	<0.01	6.94 (6.94)	<0.01	<0.01
d-limonene	<0.01	<0.01	<0.01	0.12 (0.07)	7.65 (7.23)	1.55 (0.74)	0.09 (0.05)	<0.01	<0.01	<0.01	0.03 (0.03)	0.05 (0.05)	0.27 (0.27)	0.18 (0.18)	0.03 (0.03)	<0.01
1,8-cineole	<0.01	<0.01	0.02 (0.02)	0.11 (0.07)	2.70 (2.48)	0.65 (0.27)	0.06 (0.04)	<0.01	<0.01	<0.01	0.02 (0.02)	<0.01	0.08 (0.08)	2.13 (2.20)	0.02 (0.02)	<0.01
β-Ocimene	<0.01	<0.01	<0.01	<0.01	<0.01	<0.01	<0.01	<0.01	0.04 (0.04)	<0.01	0.04 (0.04)	0.04 (0.04)	0.08 (0.08)	0.09 (0.09)	0.04 (0.04)	0.06 (0.06)
γ-Terpinene	<0.01	<0.01	<0.01	0.10 (0.06)	0.26 (0.26)	0.87 (0.66)	0.06 (0.04)	<0.01	<0.01	<0.01	<0.01	<0.01	0.09 (0.09)	0.03 (0.03)	0.02 (0.02)	<0.01
Terpinolene	<0.01	<0.01	<0.01	<0.01	<0.01	<0.01	<0.01	<0.01	<0.01	<0.01	<0.01	<0.01	<0.01	1.80 (1.80)	<0.01	<0.01
Camphor	<0.01	<0.01	<0.01	<0.01	<0.01	0.04 (0.04)	<0.01	<0.01	<0.01	<0.01	<0.01	<0.01	<0.01	0.16 (0.09)	<0.01	<0.01
Isoborneol	<0.01	<0.01	<0.01	<0.01	<0.01	<0.01	<0.01	<0.01	<0.01	<0.01	<0.01	<0.01	<0.01	0.07 (0.07)	<0.01	<0.01
Terpene-4-ol	<0.01	<0.01	<0.01	<0.01	0.10 (0.10)	<0.01	<0.01	<0.01	<0.01	<0.01	<0.01	<0.01	<0.01	0.35 (0.35)	<0.01	<0.01
α-Terpineol	<0.01	<0.01	<0.01	<0.01	0.31 (0.31)	<0.01	<0.01	<0.01	<0.01	<0.01	<0.01	<0.01	<0.01	0.94 (0.94)	<0.01	<0.01
o-Cymene	<0.01	<0.01	<0.01	0.18 (0.18)	26.68 (26.01)	3.47 (1.72)	0.20 (0.12)	<0.01	<0.01	<0.01	0.04 (0.04)	<0.01	0.36 (0.36)	13.84 (13.46)	<0.01	<0.01
Bornyl acetate	<0.01	<0.01	<0.01	<0.01	<0.01	<0.01	<0.01	<0.01	<0.01	<0.01	<0.01	<0.01	<0.01	0.24 (0.24)	<0.01	<0.01
Total MTs	<0.01	´<0.01	0.02 (0.02)	0.65 (0.39)	46.96 (43.85)	9.06 (4.32)	0.52 (0.18)	<0.01	0.04 (0.04)	<0.01	0.23 (0.13)	0.20 (0.15)	1.08 (0.88)	29.72 (28.47)	0.22 (0.11)	0.06 (0.06)
Sesquiterpenes																
Copaene	<0.01	<0.01	<0.01	<0.01	0.40 (0.40)	0.03 (0.03)	<0.01	<0.01	<0.01	<0.01	<0.01	<0.01	<0.01	0.37 (0.37)	<0.01	<0.01
β-Selinene	<0.01	<0.01	<0.01	<0.01	0.20 (0.17)	0.04 (0.04)	<0.01	<0.01	<0.01	<0.01	<0.01	<0.01	<0.01	1.22 (1.22)	<0.01	<0.01
α-Selinene	<0.01	<0.01	<0.01	<0.01	0.29 (0.29)	0.09 (0.05)	<0.01	<0.01	<0.01	<0.01	<0.01	<0.01	<0.01	1.32 (1.32)	<0.01	<0.01
Cadinene	<0.01	<0.01	<0.01	<0.01	0.14 (0.14)	<0.01	<0.01	<0.01	<0.01	<0.01	<0.01	<0.01	<0.01	1.04 (1.04)	<0.01	<0.01
Total SQTs	<0.01	<0.01	<0.01	<0.01	1.03 (1.00)	0.16 (0.12)	<0.01	<0.01	<0.01	<0.01	<0.01	<0.01	0.02 (0.02)	3.96 (3.96)	<0.01	<0.01
ORVOC																
2-methylfuran	<0.01	<0.01	<0.01	<0.01	<0.01	<0.01	<0.01	<0.01	<0.01	<0.01	<0.01	<0.01	<0.01	<0.01	<0.01	0.20 (0.20)
Benzaldehyde	<0.01	<0.01	<0.01	0.42 (0.42)	1.47 (0.95)	1.60 (0.70)	0.42 (0.42)	<0.01	<0.01	<0.01	<0.01	0.14 (0.14)	0.42 (0.27)	2.32 (1.76)	0.21 (0.21)	<0.01

Octanal	<0.01	<0.01	<0.01	<0.01	<0.01	<0.01	<0.01	<0.01	<0.01	<0.01	<0.01	<0.01	<0.01	0.96 (0.96)	<0.01	<0.01
3-methyl-1,5-dinitro-6-phenoxy-azabicyclo[3.3.1]non-6-ene	<0.01	<0.01	<0.01	<0.01	<0.01	<0.01	<0.01	<0.01	<0.01	<0.01	0.04 (0.04)	0.01 (0.01)	0.08 (0.08)	<0.01	<0.01	<0.01
Nonanal	<0.01	<0.01	<0.01	<0.01	<0.01	<0.01	<0.01	<0.01	<0.01	<0.01	0.14 (0.14)	<0.01	<0.01	2.15 (2.15)	<0.01	<0.01
2-methoxy-3-(2-methyl-1-propenyl-1)-indane	<0.01	<0.01	<0.01	<0.01	<0.01	<0.01	<0.01	<0.01	<0.01	<0.01	<0.01	<0.01	<0.01	0.13 (0.13)	<0.01	<0.01
Total ORVOC	<0.01	<0.01	<0.01	0.42 (0.42)	1.47 (0.95)	1.60 (0.70)	0.42 (0.42)	<0.01	<0.01	<0.01	0.18 (0.14)	0.15 (0.14)	0.38 (0.24)	5.83 (5.20)	0.21 (0.21)	0.20 (0.20)
Other VOCs																
Methacrylic acid methyl ester	<0.01	<0.01	<0.01	<0.01	1.35 (1.35)	1.58 (0.94)	<0.01	<0.01	<0.01	<0.01	<0.01	0.29 (0.29)	0.39 (0.39)	3.93 (2.55)	0.49 (0.49)	<0.01
Toluene	0.21 (0.03)	0.18 (0.01)	0.27 (0.10)	0.33 (0.01)	0.62 (0.27)	0.55 (0.08)	0.19 (0.06)	0.21 (0.02)	0.24 (0.03)	0.16 (0.06)	0.33 (0.06)	0.26 (0.04)	0.33 (0.04)	0.81 (0.32)	0.23 (0.03)	0.20 (0.01)
Methoxy-phenyl-oxime	<0.01	<0.01	<0.01	<0.01	0.24 (0.24)	0.22 (0.22)	<0.01	<0.01	<0.01	<0.01	<0.01	<0.01	<0.01	0.71 (0.71)	0.27 (0.27)	<0.01
Acetophenone	<0.01	<0.01	<0.01	<0.01	0.34 (0.34)	0.17 (0.17)	<0.01	<0.01	<0.01	<0.01	<0.01	<0.01	<0.01	1.17 (1.17)	<0.01	<0.01
Total other VOCs	0.21 (0.03	0.18 (0.01)	0.27 (0.10)	0.33 (0.01)	2.54 (1.89)	2.51 (0.84)	0.19 (0.06)	0.21 (0.02)	0.24 (0.03)	0.16 (0.06)	0.33 (0.06)	0.55 (0.31)	0.72 (0.38)	6.61 (4.61)	1.00 (0.48)	0.20 (0.01)
**Total BVOCs**	**0.21 (0.03)**	**0.18 (0.01)**	**0.29 (0.11)**	**1.40 (0.55)**	**53.01 (48.68)**	**14.25 (5.54)**	**1.71 (1.06)**	**0.21 (0.02)**	**0.28 (0.05)**	**0.16 (0.06)**	**0.74 (0.11)**	**1.46 (0.42)**	**8.34 (4.88)**	**53.34 (40.53)**	**2.76 (0.81)**	**0.47 (0.20)**
