# Supplementary material for: Diel Variation of Biogenic Volatile Organic Compound Emissions- A field Study in the Sub, Low and High Arctic on the Effect of Temperature and Light
Source: PLoS One. 2015 Apr 21;10(4):e0123610. doi: 10.1371/journal.pone.0123610 (PMC4405581; doi:10.1371/journal.pone.0123610)
Supplement: S6 Table — (PDF) [file pone.0123610.s006.pdf]

Table S6. Mean (SE) biogenic volatile organic compound (BVOC) emissions from a high arctic *Salix*-dominated heath in control and warming treatments (n=4) during a 24-hour period the 25-26 July.

Emission ($\mu g\ m^{-2}\ h^{-1}$)	Control								Warming							
Time	00:00	03:00	06:00	09:00	12:00	15:00	18:00	21:00	00:00	03:00	06:00	09:00	12:00	15:00	18:00	21:00
Isoprene	<0.01	<0.01	5.14 (3.78)	3.46 (2.20)	97.37 (61.22)	175.7 (66.43)	37.17 (27.22)	1.63 (0.83)	<0.01	<0.01	1.97 (0.74)	12.60 (11.06)	104.1 (43.12)	290.4 (111.2)	223.1 (60.52)	7.43 (4.30)
Monoterpenes																
1,8-cineole	<0.01	<0.01	0.02 (0.02)	<0.01	0.03 (0.03)	0.04 (0.02)	<0.01	<0.01	<0.01	<0.01	<0.01	<0.01	0.09 (0.03)	0.08 (0.05)	0.03 (0.03)	<0.01
β-Ocimene	<0.01	<0.01	<0.01	<0.01	<0.01	<0.01	<0.01	<0.01	<0.01	<0.01	<0.01	<0.01	0.08 (0.08)	0.12 (0.12)	0.04 (0.04)	<0.01
Bornyl acetate	<0.01	<0.01	<0.01	<0.01	<0.01	<0.01	<0.01	0.03 (0.03)	<0.01	<0.01	<0.01	<0.01	<0.01	<0.01	<0.01	<0.01
Total MTs	<0.01	<0.01	0.02 (0.02)	<0.01	0.03 (0.03)	0.04 (0.02)	<0.01	0.03 (0.03	<0.01	<0.01	<0.01	<0.01	0.17 (0.09)	0.20 (0.10)	0.07 (0.04)	<0.01
ORVOCs																
2-methylfuran	<0.01	<0.01	<0.01	<0.01	<0.01	<0.01	<0.01	<0.01	<0.01	<0.01	<0.01	<0.01	<0.01	<0.01	0.24 (0.24)	<0.01
Benzene	<0.01	<0.01	<0.01	0.18 (0.18)	<0.01	<0.01	0.35 (0.35)	<0.01	<0.01	<0.01	<0.01	<0.01	0.30 (0.30)	<0.01	<0.01	<0.01
Heptanal	<0.01	<0.01	<0.01	<0.01	0.15 (0.15)	<0.01	<0.01	<0.01	<0.01	<0.01	<0.01	<0.01	<0.01	<0.01	<0.01	<0.01
Benzaldehyde	<0.01	<0.01	<0.01	<0.01	0.22 (0.22)	0.18 (0.18)	0.43 (0.43)	<0.01	<0.01	<0.01	<0.01	<0.01	0.54 (0.54)	0.31 (0.31)	<0.01	0.19 (0.19)
Octanal	<0.01	<0.01	<0.01	<0.01	<0.01	<0.01	<0.01	<0.01	<0.01	<0.01	<0.01	<0.01	<0.01	<0.01	0.27 (0.27)	<0.01
2-hydroxy-benzaldehyde	<0.01	<0.01	<0.01	<0.01	<0.01	<0.01	<0.01	<0.01	<0.01	<0.01	<0.01	<0.01	0.16 (0.16)	0.07 (0.07)	<0.01	<0.01
Nonanal	<0.01	<0.01	<0.01	<0.01	<0.01	0.75 (0.75)	<0.01	<0.01	<0.01	<0.01	<0.01	<0.01	<0.01	<0.01	<0.01	<0.01
Decanal	<0.01	<0.01	<0.01	<0.01	<0.01	0.15 (0.10)	<0.01	<0.01	<0.01	<0.01	<0.01	<0.01	<0.01	0.09 (0.09)	<0.01	<0.01
3-methoxy-17-methyl-morphinan-14-ol	<0.01	<0.01	<0.01	<0.01	0.05 (0.04)	<0.01	<0.01	<0.01	<0.01	<0.01	<0.01	<0.01	<0.01	<0.01	<0.01	<0.01
Total ORVOC	<0.01	<0.01	<0.01	0.18 (0.18)	0.42 (0.35)	1.08 (0.79)	0.78 (0.78)	<0.01	<0.01	<0.01	<0.01	<0.01	1.01 (1.01)	0.46 (0.36)	0.51 (0.30)	0.19 (0.19)
Other VOCs																
2-methyl-butane	<0.01	<0.01	7.34 (7.24)	<0.01	<0.01	<0.01	<0.01	<0.01	<0.01	3.76 (3.76)	<0.01	<0.01	<0.01	<0.01	<0.01	<0.01
Methacrylic acid methyl ester	<0.01	<0.01	<0.01	0.53 (0.53)	2.30 (1.36)	4.87 (2.75)	1.38 (0.82)	0.28 (0.28)	<0.01	<0.01	<0.01	<0.01	5.19 (5.19)	8.14 (4.74)	5.28 (1.03)	0.70 (0.70)
Toluene	0.32 (0.02)	0.38 (0.03)	0.53 (0.05)	0.41 (0.03)	0.46 (0.03)	0.60 (0.06)	0.34 (0.12)	0.27 (0.09)	0.32 (0.02)	0.35 (0.03)	0.43 (0.05)	0.38 (0.02)	0.59 (0.09)	0.54 (0.07)	0.45 (0.05)	0.37 (0.03)
Methoxy-phenyl-oxime	<0.01	<0.01	<0.01	<0.01	0.05 (0.05)	0.27 (0.27)	0.68 (0.68)	<0.01	<0.01	<0.01	<0.01	<0.01	0.40 (0.40)	<0.01	<0.01	<0.01
Total other VOCs	0.32 (0.02)	0.38 (0.03)	7.87 (7.36)	0.94 (0.55)	2.80 (1.42)	5.74 (3.03)	2.39 (0.83)	0.55 (0.33)	0.32 (0.02)	4.10 (3.77)	0.43 (0.05)	0.38 (0.02)	6.60 (6.09)	8.68 (4.71)	5.74 (1.08)	1.07 (0.67)
**Total BVOCs**	**0.32 (0.02)**	**0.38 (0.03)**	**13.03 (11.10)**	**4.59 (2.71)**	**100.6 (60.39)**	**182.6 (64.28)**	**40.4 (27.43)**	**2.21 (0.73)**	**0.32 (0.02)**	**4.10 (3.77)**	**2.40 (0.71)**	**12.98 (11.06)**	**111.9 (37.54)**	**299.7 (111.5)**	**229.4 (61.57)**	**8.69 (4.01)**
