# Supplementary material for: Diel Variation of Biogenic Volatile Organic Compound Emissions- A field Study in the Sub, Low and High Arctic on the Effect of Temperature and Light
Source: PLoS One. 2015 Apr 21;10(4):e0123610. doi: 10.1371/journal.pone.0123610 (PMC4405581; doi:10.1371/journal.pone.0123610)
Supplement: S7 Table — (PDF) [file pone.0123610.s007.pdf]

Table S7. Mean (SE) biogenic volatile organic compound (BVOC) emissions from one *Betula*- and one *Salix*-dominated heath (n=4) in the Low Arctic during a 24-hour period the 18-19 June 2013.

Emission (µg m^{-2} h^{-1})	*Betula*								*Salix*							
Time	00:00	03:00	06:00	09:00	12:00	15:00	18:00	21:00	00:00	03:00	06:00	09:00	12:00	15:00	18:00	21:00
Isoprene	<0.01	<0.01	<0.01	<0.01	29.23 (18.54)	32.66 (7.27)	<0.01	<0.01	<0.01	<0.01	<0.01	3.09 (2.01)	187.68 (78.86)	408.7 (190.0)	3.34 (2.03)	<0.01
Monoterpenes																
α-thujene	<0.01	<0.01	<0.01	<0.01	1.27 (1.27)	<0.01	<0.01	<0.01	<0.01	<0.01	<0.01	<0.01	3.27 /3.27)	1.95 (1.95)	<0.01	<0.01
α-pinene	<0.01	<0.01	<0.01	<0.01	0.44 (0.22)	0.34 (0.20)	0.08 (0.08)	<0.01	<0.01	<0.01	<0.01	<0.01	0.13 (0.13)	<0.01	<0.01	<0.01
Camphene	<0.01	<0.01	<0.01	<0.01	<0.01	<0.01	<0.01	0.06 (0.06)	<0.01	<0.01	<0.01	<0.01	<0.01	<0.01	<0.01	<0.01
α-phellandrene	<0.01	<0.01	<0.01	<0.01	<0.01	0.09 (0.09)	<0.01	<0.01	<0.01	<0.01	<0.01	<0.01	<0.01	<0.01	<0.01	<0.01
Unidentified MT	<0.01	<0.01	<0.01	<0.01	<0.01	0.15 (0.15)	<0.01	<0.01	<0.01	<0.01	<0.01	<0.01	<0.01	<0.01	0.11 (0.11)	<0.01
d-limonene	<0.01	<0.01	<0.01	<0.01	0.19 (0.11)	0.49 (0.23)	<0.01	0.13 (0.13)	<0.01	<0.01	<0.01	<0.01	0.12 (0.12)	<0.01	<0.01	<0.01
Unidentified MT	<0.01	<0.01	<0.01	<0.01	0.18 (0.11)	0.67 (0.29)	0.00	0.00	<0.01	<0.01	<0.01	<0.01	<0.01	0.54 (0.43)	<0.01	<0.01
p-cymene	<0.01	<0.01	<0.01	0.17 (0.11)	0.49 (0.10)	1.19 (0.46)	0.07 (0.07)	0.33 (0.33)	<0.01	<0.01	<0.01	<0.01	0.34 (0.20)	0.77 (0.22)	<0.01	0.21 (0.21)
γ-terpinene	<0.01	<0.01	<0.01	<0.01	0.11 (0.11)	0.27 (0.20)	0.05 (0.05)	<0.01	<0.01	<0.01	<0.01	<0.01	0.12 (0.12)	0.16 (0.16)	<0.01	<0.01
β-cis-ocimene	<0.01	<0.01	<0.01	<0.01	0.12 (0.12)	0.17 (0.17)	0.00	<0.01	<0.01	<0.01	<0.01	<0.01	0.24 (0.24)	0.37 (0.37)	<0.01	<0.01
1,8-cineole	<0.01	<0.01	<0.01	0.14 (0.09)	0.88 (0.40)	1.86 (1.21)	0.11 (0.11)	0.05 (0.05)	<0.01	<0.01	<0.01	<0.01	0.64 (0.27)	0.98 (0.37)	<0.01	0.05 (0.05)
Camphor	<0.01	<0.01	<0.01	<0.01	<0.01	<0.01	<0.01	0.13 (0.13)	<0.01	<0.01	<0.01	<0.01	<0.01	<0.01	<0.01	<0.01
Bornyl acetate	<0.01	<0.01	<0.01	<0.01	<0.01	<0.01	<0.01	0.11 (0.11)	<0.01	<0.01	<0.01	<0.01	0.03 (0.03)	<0.01	<0.01	0.04 (0.04)
Total MTs	<0.01	<0.01	<0.01	0.37 (0.22)	3.68 (1.95)	5.24 (2.59)	0.31 (0.22)	0.81 (0.81)	<0.01	<0.01	<0.01	<0.01	4.89 (4.28)	4.88 (2.99)	<0.01	0.29 (0.29)
Sesquiterpenes																
Caryophyllene	<0.01	<0.01	<0.01	1.24 (0.54)	20.50 (8.75)	27.63 (10.66)	0.43 (0.43)	<0.01	<0.01	<0.01	<0.01	<0.01	3.37 (2.51)	4.02 (3.14)	<0.01	<0.01
Allo-Aromadendrene	<0.01	<0.01	<0.01	<0.01	<0.01	0.68 (0.48)	<0.01	<0.01	<0.01	<0.01	<0.01	<0.01	<0.01	<0.01	<0.01	<0.01
α-caryophyllene	<0.01	<0.01	<0.01	0.21 (0.12)	2.68 (1.18)	4.48 (1.95)	0.08 (0.08)	<0.01	<0.01	<0.01	<0.01	<0.01	0.13 (0.13)	0.25 (0.16)	<0.01	<0.01
α-farnesene	<0.01	<0.01	<0.01	<0.01	<0.01	<0.01	<0.01	<0.01	<0.01	<0.01	<0.01	<0.01	0.10 (0.10)	0.20 (0.20)	<0.01	<0.01
Total SQTs	<0.01	<0.01	<0.01	1.44 (0.64)	23.18 (9.88)	32.80 (12.97)	0.50 (050)	<0.01	<0.01	<0.01	<0.01	<0.01	3.60 (2.74)	4.47 (3.50)	<0.01	<0.01
ORVOCs																
2-methylfuran	<0.01	<0.01	<0.01	<0.01	1.44 (0.73)	2.77 (1.00)	<0.01	<0.01	<0.01	<0.01	<0.01	<0.01	<0.01	1.74 (1.01)	<0.01	<0.01
(z,z)-2,4-hexadiene	<0.01	<0.01	<0.01	<0.01	<0.01	1.10 (1.10)	<0.01	<0.01	<0.01	<0.01	<0.01	<0.01	0.66 (0.66)	0.82 (0.82)	<0.01	<0.01
2-methyl-1,3-pentadiene	<0.01	<0.01	<0.01	<0.01	1.03 (1.03)	<0.01	<0.01	<0.01	<0.01	<0.01	<0.01	<0.01	<0.01	<0.01	<0.01	<0.01
Benzene	<0.01	<0.01	<0.01	<0.01	0.36 (0.36)	1.63 (0.69)	0.22 (0.22)	0.42 (0.42)	<0.01	<0.01	<0.01	1.67 (1.06)	<0.01	2.12 (0.53)	<0.01	0.06 (0.06)
1-octene	<0.01	<0.01	<0.01	<0.01	<0.01	0.13 (0.13)	<0.01	<0.01	<0.01	<0.01	<0.01	<0.01	<0.01	<0.01	<0.01	<0.01
2-hexenal	<0.01	<0.01	<0.01	<0.01	0.19 (0.19)	0.72 (0.72)	<0.01	<0.01	<0.01	<0.01	<0.01	<0.01	0.62 (0.62)	<0.01	<0.01	0.05 (0.05)
Cyclooctatetraene	<0.01	<0.01	<0.01	<0.01	<0.01	<0.01	<0.01	<0.01	<0.01	<0.01	<0.01	<0.01	0.09 (0.09)	0.13 (0.13)	<0.01	<0.01
Benzaldehyde	<0.01	<0.01	<0.01	0.25 (0.25)	1.34 (0.77)	3.41 (0.85)	0.30 (0.30)	0.17 (0.16)	<0.01	<0.01	<0.01	0.26 (0.16)	1.96 (1.38)	2.34 (1.58)	0.42 (0.24)	<0.01

2-methyl-,exo-bicyclo[2.2.1]heptane	<0.01	<0.01	<0.01	<0.01	0.21 (0.21)	1.37 (1.37)	<0.01	<0.01	<0.01	<0.01	<0.01	<0.01	<0.01	<0.01	<0.01	0.03 (0.03)
Total ORVOCs	<0.01	<0.01	<0.01	0.25 (0.25)	4.26 (2.27)	11.13 (3.42)	0.32 (0.29)	0.58 (0.17)	<0.01	<0.01	<0.01	1.93 (1.22)	3.33 (2.66)	7.19 (3.64)	0.42 (0.24)	0.15 (0.15)
Other VOCs																
Cyclopentane	20.91 (20.91)	56.89 (56.89)	<0.01	5.32 (5.32	<0.01	<0.01	<0.01	8.28 (8.28)	36.35 (23.21)	<0.01	42.32 (42.32)	38.38 (38.38)	<0.01	<0.01	<0.01	17.04 (17.04)
Methylbutane	64.77 (52.62)	246.3 (221.4)	99.9 (49.35)	307.3 (190.4)	74.12 (65.27)	24.73 (21.61)	12.98 (7.52)	41.74 (32.50)	121.8 (62.53)	88.52 (54.87)	284.6 (181.4)	268.6 (226.0)	50.95 (36.26)	15.96 (15.96)	8.15 (8.15)	50.05 (47.63)
p-xylene	<0.01	<0.01	<0.01	<0.01	<0.01	<0.01	<0.01	<0.01	<0.01	<0.01	<0.01	<0.01	0.13 (0.13)	<0.01	<0.01	<0.01
Methoxy-phenyl-oxime	<0.01	<0.01	<0.01	<0.01	1.70 (1.06)	2.13 (1.23)	<0.01	<0.01	<0.01	<0.01	<0.01	0.79 (0.79)	0.94 (0.94)	0.97 (0.97)	<0.01	<0.01
Acetophenone	<0.01	<0.01	<0.01	<0.01	1.96 (1.96)	3.32 (1.99)	0.40 (0.40)	<0.01	<0.01	<0.01	<0.01	<0.01	3.12 (1.94)	2.95 (1.99)	<0.01	<0.01
Total Other VOCs	85.69 (73.48)	303.2 (278.2)	99.89 (49.35)	312.6 (195.6)	77.78 (66.68)	30.17 (22.52)	13.38 (7.78)	50.01 (23.90)	158.2 (85.45)	88.52 (54.87)	326.9 (223.4)	307.8 (264.1)	55.14 (37.54)	19.88 (14.88)	8.15 (8.15)	67.09 (64.67)
**Total BVOCs**	**85.69 (73.48)**	**303.2 (278.2)**	**99.89 (49.35)**	**314.7 (195.1)**	**138.1 (94.34)**	**112.0 (20.07)**	**14.52 (7.7)**	**51.40 (23.79)**	**158.2 (85.45)**	**88.52 (54.87)**	**326.9 (223.4)**	**312.8 (262.4)**	**254.7 (60.32)**	**445.1 (204.3)**	**11.91 (7.10)**	**67.53 (64.78)**
