# Supplementary material for: Diel Variation of Biogenic Volatile Organic Compound Emissions- A field Study in the Sub, Low and High Arctic on the Effect of Temperature and Light
Source: PLoS One. 2015 Apr 21;10(4):e0123610. doi: 10.1371/journal.pone.0123610 (PMC4405581; doi:10.1371/journal.pone.0123610)
Supplement: S8 Table — (PDF) [file pone.0123610.s008.pdf]

Table S8. Mean (SE) biogenic volatile organic compound (BVOC) emissions from a subarctic peatland (n=4) during a 24-hour period the 23-24 of July 2008.

Emission (µg m-2 h-1)	July											
Time	01:00	03:00	05:00	07:00	09:00	11:00	13:00	15:00	17:00	19:00	21:00	23:00
Isoprene	3.95 (3.95)	11.96 (5.96)	37.63 (16.35)	18.61 (7.06)	27.05 (7.32)	51.67 (18.91)	15.97 (15.97)	93.08 (67.96)	21.15 (13.66)	14.21 (12.75)	47.40 (29.57)	17.76 (9.55)
Monoterpinoids												
α-pinene	35.97 (4.39)	36.27 (5.98)	16.35 (28.94)	24.49 (3.73)	22.28 (1.91)	23.19 (9.74)	12.88 (5.72)	15.44 (5.10)	17.33 (2.87)	49.67 (7.43)	90.25 (26.32)	60.12 (7.37)
Camphene	1.51 (0.88)	1.74 (0.60)	0.89 (0.54)	0.41 (0.25)	0.82 (0.54)	1.65 (1.65)	<0.01	<0.01	0.26 (0.26)	0.90 (0.52)	3.39 (0.87)	2.19 (0.83)
β-pinene	<0.01	<0.01	<0.01	<0.01	<0.01	<0.01	<0.01	4.98 (4.98)	4.21 (4.21)	<0.01	<0.01	<0.01
β-myrcene	<0.01	<0.01	<0.01	<0.01	1.21 (0.80)	<0.01	<0.01	<0.01	2.52 (2.52)	<0.01	<0.01	<0.01
Carene	13.58 (0.91)	12.97 (2.05)	3.70 (2.66)	6.95 (0.97)	6.82 (2.64)	2.60 (1.75)	1.81 (1.25)	3.36 (1.23)	0.54 (0.54)	12.77 (2.79)	16.81 (6.05)	16.20 (2.24)
d-limonene	6.07 (0.47)	5.43 (0.86)	2.68 (1.12)	6.86 (5.32)	6.55 (1.34)	7.54 (1.47)	1.69 (0.77)	3.44 (2.02)	12.88 (4.06)	12.75 (6.48)	10.44 (5.53)	7.82 (3.52)
Terpinolene	0.98 (0.57)	2.01 (0.77)	0.24 (0.24)	<0.01	0.80 (0.49)	0.35 (0.22)	0.33 (0.33)	2.14 (1.24)	1.08 (1.08)	<0.01	<0.01	0.60 (0.60)
γ-terpinene	<0.01	<0.01	<0.01	<0.01	50.28 (50.10)	38.38 (37.99)	<0.01	120.5 (71.85)	<0.01	<0.01	0.68 (0.68)	<0.01
Camphor	<0.01	<0.01	<0.01	<0.01	1.02 (0.72)	<0.01	<0.01	0.27 (0.27)	<0.01	<0.01	<0.01	<0.01
1,8-cineole	<0.01	<0.01	<0.01	<0.01	0.72 (0.47)	0.22 (0.22)	<0.01	<0.01	<0.01	<0.01	<0.01	0.31 (0.31)
Linalool	<0.01	<0.01	<0.01	<0.01	0.83 (0.83)	21.93 (21.93)	1.04 (1.04)	11.63 (11.63)	<0.01	<0.01	<0.01	<0.01
Borneol	<0.01	<0.01	<0.01	<0.01	<0.01	<0.01	<0.01	0.95 (0.95)	<0.01	<0.01	<0.01	<0.01
O-cymene	<0.01	<0.01	<0.01	1.55 (1.55)	4.09 (1.94)	3.43 (2.07)	1.34 (1.34)	0.81 (0.81)	5.29 (3.22)	2.01 (2.01)	9.25 (5.84)	4.12 (2.69)
Total MTs	58.12 (5.99)	58.61 (7.63)	36.46 (6.59)	40.27 (10.92)	95.50 (55.48)	99.27 (51.37)	19.09 (9.72)	172.53 (87.01)	44.10 (4.77)	78.10 (15.46)	130.82 (43.45)	91.35 (13.18)
Sesquiterpenes												
Longifolene	0.51 (0.37)	<0.01	<0.01	<0.01	0.61 (0.61)	<0.01	<0.01	<0.01	0.81 (0.81)	<0.01	<0.01	0.60 (0.55)
trans-b-farnesene	<0.01	<0.01	<0.01	<0.01	0.30 (0.30)	0.65 (0.52)	0.90 (0.90)	<0.01	<0.01	<0.01	<0.01	<0.01
Caryophyllene	17.59 (9.28)	4.93 (2.11)	7.72 (1.42)	6.62 (1.90)	8.09 (3.26)	28.57 (11.85)	7.90 (5.13)	31.85 (7.50)	12.84 (4.14)	4.32 (2.77)	3.48 (2.01)	5.43 (2.38)
Total SQT	18.10 (9.18)	4.93 (2.11)	7.72 (1.42)	6.62 (1.90)	9.00 (3.86)	29.21 (12.26)	8.81 (5.33)	31.85 (7.50)	13.65 (4.81)	4.32 (2.77)	3.48 (2.01)	6.03 (2.26)
ORVOCs												
trans cyclohexane 1-methyl-4-(1-methylethyl)	<0.01	<0.01	<0.01	<0.01	7.74 (5.71)	4.18 (4.18)	<0.01	<0.01	13.25 (13.25)	<0.01	<0.01	<0.01
2-methylfuran	<0.01	<0.01	<0.01	<0.01	<0.01	<0.01	0.47 (0.47)	3.11 (2.04)	<0.01	<0.01	<0.01	<0.01
1,2-Pentadiene	<0.01	<0.01	<0.01	4.07 (4.07)	12.71 (8.32)	18.31 (10.69)	6.88 (6.88)	30.01 (12.90)	12.53 (7.88)	2.84 (2.84)	4.19 (4.19)	7.84 (7.84)
1-heptene	8.78 (6.83)	10.30 (10.30)	<0.01	20.25 (11.81)	3.80 (2.87)	2.33 (2.33)	4.55 (4.55)	<0.01	<0.01	<0.01	8.96 (5.40)	<0.01
3-heptene	<0.01	37.19 (14.32)	8.61 (5.28)	<0.01	12.29 (12.29)	4.69 (4.69)	21.43 (14.72)	4.52 (4.52)	3.11 (3.11)	<0.01	5.27 (5.27)	28.44 (19.49)
(e)-2-heptene	27.53 (10.47)	40.51 (10.10)	16.82 (10.87)	14.91 (8.61)	16.01 (8.04)	38.75 (28.86)	11.86 (11.86)	26.93 (9.72)	27.33 (9.99)	3.91 (3.91)	3.61 (3.61)	34.16 (21.03)
Heptanal	<0.01	<0.01	<0.01	<0.01	<0.01	<0.01	<0.01	91.21 (91.21)	<0.01	<0.01	<0.01	<0.01
Benzaldehyde	<0.01	0.34 (0.34)	<0.01	1.60 (1.60)	0.04 (0.04)	1.53 (1.53)	<0.01	0.03 (0.03)	5.45 (5.45)	<0.01	<0.01	<0.01
p-xylene	<0.01	2.75 (2.75)	<0.01	<0.01	1.20 (1.20)	<0.01	<0.01	<0.01	<0.01	<0.01	<0.01	<0.01
1-octene	<0.01	<0.01	<0.01	<0.01	<0.01	6.44 (4.13)	<0.01	<0.01	<0.01	<0.01	<0.01	<0.01
Octanal	<0.01	<0.01	<0.01	<0.01	1.56 (1.56)	<0.01	<0.01	147.96 (147.96)	1.04 (1.04)	<0.01	<0.01	<0.01
Total ORVOC	36.31 (5.57)	91.10 (25.06)	67.24 (25.43)	40.84 (19.17)	55.34 (25.06)	208.0 (188.2)	45.19 (37.17)	303.8 (255.1)	62.71 (10.85)	6.75 (6.75)	20.02 (12.29)	70.44 (42.24)
Other VOCs												
Toluene	36.17 (21.04)	78.32 (30.48)	59.09 (23.68)	62.84 (25.35)	97.13 (43.85)	82.37 (14.68)	21.72 (21.72)	13.57 (13.57)	32.45 (32.45)	38.79 (38.79)	36.82 (5.54)	46.45 (9.04)
Methoxy-phenyl-oxime	76.39 (75.39)	236.6 (115.1)	261.2 (92.60)	169.9 (98.4)	147.1 (57.37)	180.5 (180.5)	77.88 (75.74)	77.07 (77.07)	118.3 (118.3)	242.2 (196.0)	292.5 (176.8)	273.6 (118.8)
Total Other VOC	112.6 (65.71)	314.9 (106.76)	320.3 (99.09)	232.7 (92.91)	244.23 (80.62)	262.9 (194.0)	99.60 (71.11)	90.64 (73.67)	150.8 (107.6)	281.0 (196.9)	329.4 (179.9)	320.1 (121.1)
**Total BVOC**	**229.1 (74.82)**	**481.5 (125.4)**	**427.5 (95.52)**	**339.1 (86.4)**	**431.1 (100.7)**	**519.3 (157.9)**	**188.7 (69.87)**	**691.9 (318.8)**	**292.4 (121.3)**	**384.3 (198.2)**	**533.1 (216.6)**	**505.7 (128.9)**
