# Supplementary material for: Diel Variation of Biogenic Volatile Organic Compound Emissions- A field Study in the Sub, Low and High Arctic on the Effect of Temperature and Light
Source: PLoS One. 2015 Apr 21;10(4):e0123610. doi: 10.1371/journal.pone.0123610 (PMC4405581; doi:10.1371/journal.pone.0123610)
Supplement: S9 Table — (PDF) [file pone.0123610.s009.pdf]

Table S9. Mean (SE) biogenic volatile organic compound (BVOC) emissions from a subarctic peatland (n=4) during a 24-hour period the 5-6 of August 2008.

Emission (µg m^{-2} h^{-1})	August											
Time	01:00	03:00	05:00	07:00	09:00	11:00	13:00	15:00	17:00	19:00	21:00	23:00
Isoprene	<0.01	45.67 (22.21)	33.50 (24.54)	20.15 (5.06)	21.05 (6.53)	22.97 (3.76)	323.0 (171.7)	167.2 (124.8)	182.2 (61.47)	218.3 (142.2)	20.13 (20.13)	81.0 (81.0)
Monoterpinoids												
α-pinene	99.81 (33.85)	181.0 (18.93)	140.70 (14.62)	92.16 (13.55)	50.09 (7.56)	24.92 (4.93)	97.07 (35.90)	19.09 (2.02)	30.24 (4.04)	36.87 (9.65)	127.4 (14.54)	144.7 (20.36)
Camphene	4.81 (1.69)	6.57 (2.42)	6.31 (0.16)	4.24 (0.27)	0.93 (0.47)	<0.01	2.04 (1.28)	<0.01	<0.01	<0.01	4.63 (1.61)	5.86 (1.04)
Sabinene	3.34 (3.34)	5.02 (5.02)	5.54 (3.20)	3.28 (1.90)	0.56 (0.56)	<0.01	<0.01	<0.01	<0.01	<0.01	2.80 (2.80)	5.49 (3.39)
β-myrcene	0.63 (0.63)	2.22 (1.28)	1.67 (0.97)	<0.01	<0.01	<0.01	0.24 (0.24)	<0.01	<0.01	<0.01	1.96 (1.34)	0.60 (0.60)
Carene	25.55 (8.87)	44.48 (4.73)	36.48 (6.50)	23.23 (5.92)	10.90 (1.79)	1.27 (1.27)	14.88 (9.86)	1.88 (1.09)	2.97 (1.76)	4.58 (2.69)	27.40 (4.05)	36.97 (5.59)
d-limonene	16.65 (7.64)	13.52 (4.55)	17.67 (2.34)	8.08 (2.75)	3.70 (1.48)	2.18 (1.28)	7.83 (1.71)	4.63 (2.72)	7.92 (7.01)	3.51 (2.35)	14.04 (2.12)	13.05 (4.87)
Terpinolene	1.24 (1.24)	7.06 (5.41)	2.37 (1.48)	0.76 (0.76)	<0.01	<0.01	<0.01	<0.01	<0.01	1.97 (1.97)	1.96 (1.13)	5.03 (3.82)
γ-terpinene	<0.01	52.44 (52.44)	80.03 (46.21)	<0.01	0.09 (0.09)	0.52 (0.52)	0.70 (0.70)	<0.01	79.52 (79.52)	49.75 (49.75)	65.93 (64.06)	<0.01
Camphor	<0.01	<0.01	<0.01	<0.01	0.54 (0.54)	<0.01	<0.01	<0.01	<0.01	<0.01	<0.01	<0.01
1,8-cineole	<0.01	<0.01	<0.01	<0.01	0.39 (0.39)	<0.01	<0.01	<0.01	<0.01	<0.01	<0.01	1.98 (1.98)
Linalool	<0.01	0.04 (0.04)	<0.01	<0.01	<0.01	<0.01	<0.01	<0.01	<0.01	<0.01	<0.01	<0.01
O-cymene	<0.01	<0.01	4.33 (4.33)	<0.01	<0.01	<0.01	<0.01	<0.01	<0.01	<0.01	<0.01	1.07 (1.07)
Total MTs	152.0 (50.99)	312.3 (72.44)	295.1 (30.18)	131.8 (19.84)	67.20 (10.52)	28.90 (6.51)	122.8 (48.17)	25.60 (3.64)	120.6 (79.17)	46.92 (12.58)	246.1 (78.01)	264.5 (63.23)
Sesquiterpenes												
Longifolene	<0.01	<0.01	<0.01	<0.01	0.22 (0.22)	<0.01	<0.01	<0.01	<0.01	<0.01	<0.01	<0.01
α-patchoulene	<0.01	1.03 (1.03)	<0.01	<0.01	<0.01	<0.01	<0.01	<0.01	<0.01	<0.01	<0.01	<0.01
Caryophyllene	1.66 (1.07)	1.07 (1.07)	0.38 (0.38)	0.34 (0.34)	2.81 (1.32)	3.49 (2.26)	2.87 (2.87)	<0.01	0.61 (0.61)	6.32 (3.02)	2.34 (1.02)	2.57 (1.96)
Total SQTs	1.66 (1.07)	2.10 (1.21)	0.38 (0.38)	0.34 (0.34)	3.03 (1.47)	3.49 (2.26)	2.87 (2.87)	<0.01	0.61 (0.61)	6.32 (3.02)	2.34 (1.02)	2.57 (1.96)
ORVOCs												
trans-cyclohexane 1-methyl-4-(1-methylethyl)	<0.01	<0.01	<0.01	<0.01	1.34 (1.34)	<0.01	<0.01	<0.01	<0.01	<0.01	<0.01	<0.01
2-methylfuran	<0.01	<0.01	<0.01	<0.01	1.06 (1.06)	<0.01	<0.01	<0.01	<0.01	<0.01	<0.01	<0.01
1,2-Pentadiene	7.49 (4.34)	20.86 (10.34)	<0.01	<0.01	<0.01	15.76 (9.33)	<0.01	54.10 (46.81)	<0.01	19.49 (19.49)	18.63 (11.58)	9.14 (5.72)
Hexane	<0.01	<0.01	<0.01	<0.01	<0.01	<0.01	5.61 (5.61)	<0.01	<0.01	<0.01	<0.01	<0.01
1-heptene	<0.01	<0.01	7.25 (7.25)	<0.01	12.15 (6.54)	<0.01	5.71 (5.71)	51.60 (17.72)	<0.01	19.46 (14.66)	2.91 (2.91)	<0.01
3-heptene	49.35 (21.43)	<0.01	14.15 (8.19)	<0.01	7.79 (5.55)	<0.01	7.38 (7.38)	14.69 (9.82)	<0.01	45.76 (28.11)	28.65 (17.16)	8.84 (8.84)
(e)-2-heptene	52.55 (20.22)	13.79 (8.83)	18.33 (6.22)	0.00	23.58 (14.50)	15.51 (9.58)	6.76 (6.76)	40.25 (15.40)	6.77 (4.56)	45.19 (31.44)	62.13 (20.85)	34.97 (6.33)
Benzaldehyde	0.17 (0.17)	2.62 (2.09)	<0.01	<0.01	0.66 (0.66)	<0.01	<0.01	2.86 (2.86)	<0.01	1.63 (1.63)	3.72 (3.72)	0.49 (0.49)
(z)-acetate- 3-hexen-1-ol	<0.01	<0.01	<0.01	<0.01	<0.01	<0.01	<0.01	<0.01	<0.01	<0.01	8.74 (8.74)	<0.01
1-octene	<0.01	<0.01	<0.01	<0.01	2.97 (2.97)	3.38 (3.38)	<0.01	2.31 (2.31)	<0.01	<0.01	1.81 (1.81)	<0.01
Total ORVOCs	109.6 (45.51)	37.27 (12.45)	39.73 (18.69)	<0.01	49.54 (19.11)	34.65 (12.43)	25.47 (10.74)	165.8 (84.80)	6.77 (4.56)	131.5 (56.43)	126.6 (30.90)	53.44 (11.73)
Other VOCs												
Toluene	37.34 (13.07)	12.66 (12.66)	33.31 (11.50)	15.26 (10.09)	7.45 (5.47)	18.60 (10.75)	27.15 (9.58)	<0.01	<0.01	9.25 (9.25)	56.28 (5.95)	30.16 (10.28)
Methoxy-phenyl-oxime	3.57 (3.57)	61.43 (61.43)	120.35 (71.03)	290.1 (253.0)	319.7 (185.2)	228.1 (228.1)	29.27 (21.34)	31.48 (31.48)	81.68 (81.68)	16.60 (8.86)	404.3 (371.9)	51.50 (34.44)
Total Other VOCs	40.91 (14.84)	74.09 (58.44)	153.66 (77.83)	305.4 (248.0)	327.1 (184.9)	246.7 (222.0)	56.42 (28.54)	31.48 (31.48)	81.68 (81.68)	25.84 (14.77)	460.6 (377.2)	81.66 (41.57)
**Total BVOCs**	**304.2 (105.3)**	**471.5 (43.3)**	**522.4 (48.4)**	**457.6 (245.2)**	**467.9 (179.3)**	**336.7 (234.2)**	**530.5 (124.74)**	**390.1 (106.9)**	**391.9 (109.9)**	**428.9 (158.1)**	**855.8 (320.6)**	**483.2 (123.0)**
