# Supplementary material for: Diel Variation of Biogenic Volatile Organic Compound Emissions- A field Study in the Sub, Low and High Arctic on the Effect of Temperature and Light
Source: PLoS One. 2015 Apr 21;10(4):e0123610. doi: 10.1371/journal.pone.0123610 (PMC4405581; doi:10.1371/journal.pone.0123610)
Supplement: S10 Table — (PDF) [file pone.0123610.s010.pdf]

Table S10. Mean (SE) biogenic volatile organic compound (BVOC) emissions under light and dark conditions in control (C) and warming (W) treatment (n=4) from a low arctic heath.

Emission ($\mu g\ m^{-2}\ h^{-1}$)	Treatment	C	C	W	W
	light/dark	Light	Dark	Light	Dark
	Isoprene	1.43 (1.43)	<0.01	4.13 (2.65)	<0.01
Monoterpenes					
	α-thujene	<0.01	<0.01	0.79 (0.79)	<0.01
	α-pinene	<0.01	0.11 (0.11)	0.09 (0.09)	0.13 (0.08)
	α-fenchene	<0.01	<0.01	1.01 (0.52)	<0.01
	Camphene	0.79 (0.36)	0.17 (0.11)	1.53 (1.40)	0.67 (0.23)
	β-pinene	<0.01	<0.01	0.02 (0.02)	<0.01
	α-phellandrene	0.07 (0.07)	<0.01	0.08 (0.08)	<0.01
	3-carene	0.24 (0.24)	<0.01	0.87 (0.30)	<0.01
	d-limonene	1.98 (1.22)	0.18 (0.18)	2.08 (2.01)	0.94 (0.37)
	Terpinene	0.14 (0.14)	<0.01	0.88 (0.33)	<0.01
	p-cymene	7.84 (5.27)	1.20 (0.77)	6.25 (1.39)	1.75 (0.54)
	Terpinolene	<0.01	<0.01	1.71 (1.07)	1.04 (1.04)
	1,8-cineole	1.07 (1.07)	<0.01	4.64 (2.86)	1.09 (0.88)
	Fenchol	<0.01	<0.01	0.22 (0.13)	<0.01
	Terpinene-4-ol	0.11 (0.11)	<0.01	0.54 (0.33)	0.16 (0.16)
	Total MTs	12.24 (6.95)	1.66 (1.16)	20.71 (8.12)	5.79 (2.68)
Sesquiterpenes					
	Copaene	0.16 (0.16)	<0.01	<0.01	<0.01
	β-Bourbonene	0.16 (0.16)	<0.01	<0.01	<0.01
	β-Guaiene	<0.01	<0.01	1.42 (0.66)	<0.01
	α-Selinene	<0.01	<0.01	1.25 (0.73)	<0.01
	Valencene	<0.01	<0.01	2.02 (0.76)	0.15 (0.15)
	δ-cadinene	<0.01	<0.01	0.40 (0.25)	0.02 (0.02)
	3,3,7,7-tetramethyl-5-(2-methyl-1-propenyl)-tricyclo[4.1.0.0(2,4)]heptane	<0.01	<0.01	0.39 (0.39)	<0.01
	β-panasinsene	<0.01	<0.01	0.69 (0.69)	<0.01
	Total SQTs	0.32 (0.18)	<0.01	6.17 (3.05)	0.17 (0.17)
ORVOCs					
	2-methylfuran	0.57 (0.57)	<0.01	1.89 (1.89)	1.01 (1.01)
	Benzene	5.28 (3.08)	2.86 (1.90)	3.31 (1.94)	2.19 (1.28)
	Methyl 2-methylpropenoate	3.10 (1.84)	3.94 (3.94)	3.35 (3.35)	12.86 (10.81)
	1-octene	0.15 (0.15)	<0.01	1.12 (1.12)	<0.01
	Hexanal	7.08 (7.08)	8.32 (8.32)	24.64 (24.64)	24.77 (10.01)
	o-xylene	1.78 (0.69)	0.55 (0.27)	2.86 (0.95)	2.29 (0.76)
	Heptanal	5.50 (1.84)	<0.01	3.20 (3.20)	3.85 (2.27)
	Benzaldehyde	10.06 (5.70)	7.78 (4.05)	13.27 (7.94)	3.49 (3.37)
	2-pentylfuran	<0.01	0.29 (0.29)	0.87 (0.87)	<0.01
	Octanal	7.68 (2.57)	<0.01	13.41 (5.63)	3.72 (2.36)

	Benzeneacetaldehyde	0.51 (0.51)	<0.01	<0.01	<0.01
	Nonanal	1.01 (1.01)	<0.01	17.37 (10.74)	1.62 (1.62)
	Total ORVOCs	42.79 (16.75)	23.76 (17.34)	85.29 (45.73)	55.81 (24.29)
Other VOCs					
	2-methyl-butane	<0.01	4.26 (2.52)	4.22 (4.22)	2.68 (2.68)
	Toluene	4.40 (0.63)	2.43 (1.00)	9.15 (2.27)	5.46 (0.36)
	p-xylene	0.42 (0.25)	0.18 (0.12)	0.49 (0.49)	0.10 (0.10)
	Acetophenone	2.64 (2.64)	<0.01	4.43 (4.43)	0.57 (0.57)
	Total Other VOCs	7.46 (3.30)	6.86 (2.02)	18.28 (5.76)	8.80 (2.60)
**Total BVOCs**		**64.18 (25.86)**	**32.28 (16.31)**	**134.48 (62.20)**	**70.48 (16.97)**
